# Mitochondrial DNA signals driving immune responses: Why, How, Where?

**DOI:** 10.1186/s12964-025-02042-0

**Published:** 2025-04-22

**Authors:** Luca Giordano, Sarah A. Ware, Claudia J. Lagranha, Brett A. Kaufman

**Affiliations:** 1 Center for Metabolism and Mitochondrial Medicine, Division of Cardiology, Department of Medicine, University of Pittsburgh, Pittsburgh, PA USA; 2 Heart, Lung, and Blood Vascular Medicine Institute, University of Pittsburgh, Pittsburgh, PA USA; 3 Universities of Giessen and Marburg Lung Center (UGMLC), Member of the German Center for Lung Research (DZL), Cardio-Pulmonary Institute (CPI), Justus-Liebig-University, Giessen, Germany

**Keywords:** Mitochondria, Mitochondrial DNA, Circulating cell-free DNA, DNA-sensing receptors, Inflammation, Innate immunity

## Abstract

There has been a recent expansion in our understanding of DNA-sensing mechanisms. Mitochondrial dysfunction, oxidative and proteostatic stresses, instability and impaired disposal of nucleoids cause the release of mitochondrial DNA (mtDNA) from the mitochondria in several human diseases, as well as in cell culture and animal models. Mitochondrial DNA mislocalized to the cytosol and/or the extracellular compartments can trigger innate immune and inflammation responses by binding DNA-sensing receptors (DSRs). Here, we define the features that make mtDNA highly immunogenic and the mechanisms of its release from the mitochondria into the cytosol and the extracellular compartments. We describe the major DSRs that bind mtDNA such as cyclic guanosine-monophosphate-adenosine-monophosphate synthase (cGAS), Z-DNA-binding protein 1 (ZBP1), NOD-, LRR-, and PYD- domain-containing protein 3 receptor (NLRP3), absent in melanoma 2 (AIM2) and toll-like receptor 9 (TLR9), and their downstream signaling cascades. We summarize the key findings, novelties, and gaps of mislocalized mtDNA as a driving signal of immune responses in vascular, metabolic, kidney, lung, and neurodegenerative diseases, as well as viral and bacterial infections. Finally, we define common strategies to induce or inhibit mtDNA release and propose challenges to advance the field.

## Mitochondrial origin and functions

Mitochondria are semi-autonomous organelles located in the cytoplasm of eukaryotic cells. They are derived from an endosymbiotic event between a facultative anaerobic α-proteobacterium—most probably belonged to the order of Rickettsiales—and a host cell Asgard Archaea, approximately 1.45 billion years ago [1]. This event enabled the α-proteobacterium to respire and produce adenosine 5’-triphosphate (ATP) [1, 2]. Mitochondria have two membranes that may be the result of that endosymbiotic event—an outer mitochondrial membrane (OMM) that physically separates the cytoplasm from the intermembrane space (IMS) and an inner mitochondrial membrane (IMM) that borders the mitochondrial matrix. The IMM forms convoluted pleomorphic invaginations (*i.e.*, cristae) and houses the electron transport system (ETS). The ETS consists of four mitochondrial complexes—NADH-ubiquinone oxidoreductase (complex I; cI), succinate-ubiquinone oxidoreductase (complex II; cII), ubiquinol-cytochrome c oxidoreductase (complex III; cIII), and the cytochrome c oxidase (complex IV; cIV)—and two electron carriers, coenzyme Q (CoQ, ubiquinone) and cytochrome *c* (cyt* c*) [3]*.* Electrons enter the ETS through reduced nicotinamide adenine dinucleotide (NADH) and succinate, being oxidized by cI and cII, respectively. Electrons are then transferred by CoQ, cyt *c*, and cIII, to cIV, where molecular oxygen is reduced to water. In this process, cI, cIII, and cIV generate an electrochemical difference (∆p) by pumping hydrogen ions into the IMS, establishing and maintaining the pH gradient (∆pH) and the mitochondrial membrane potential (∆Ψ_mt_). ATP synthase (complex V; cV) uses the energy of the ∆p to phosphorylate ADP with inorganic phosphate (Pi) and produces ATP, coupling the oxygen consumption to oxidative phosphorylation (OXPHOS) [3].

The mitochondrion’s primary role is frequently thought of as bioenergetic—it synthesizes ATP. However, mitochondria are multifunctional organelles involved in catabolic and anabolic pathways [3], oxygen sensing [4], pyrimidine synthesis [5, 6], redox homeostasis [7, 8], stem cell differentiation [9, 10], senescence and aging [9, 11], cell death [12, 13], and immunity [14–16]. In the following section, we will focus specifically on the biology of mitochondrial DNA (mtDNA), which is essential to OXPHOS but plays a role in immune signaling, contributing to the pathophysiology of several diseases.

## Organization and content of the human mitochondrial genome

**Fig. 1:**
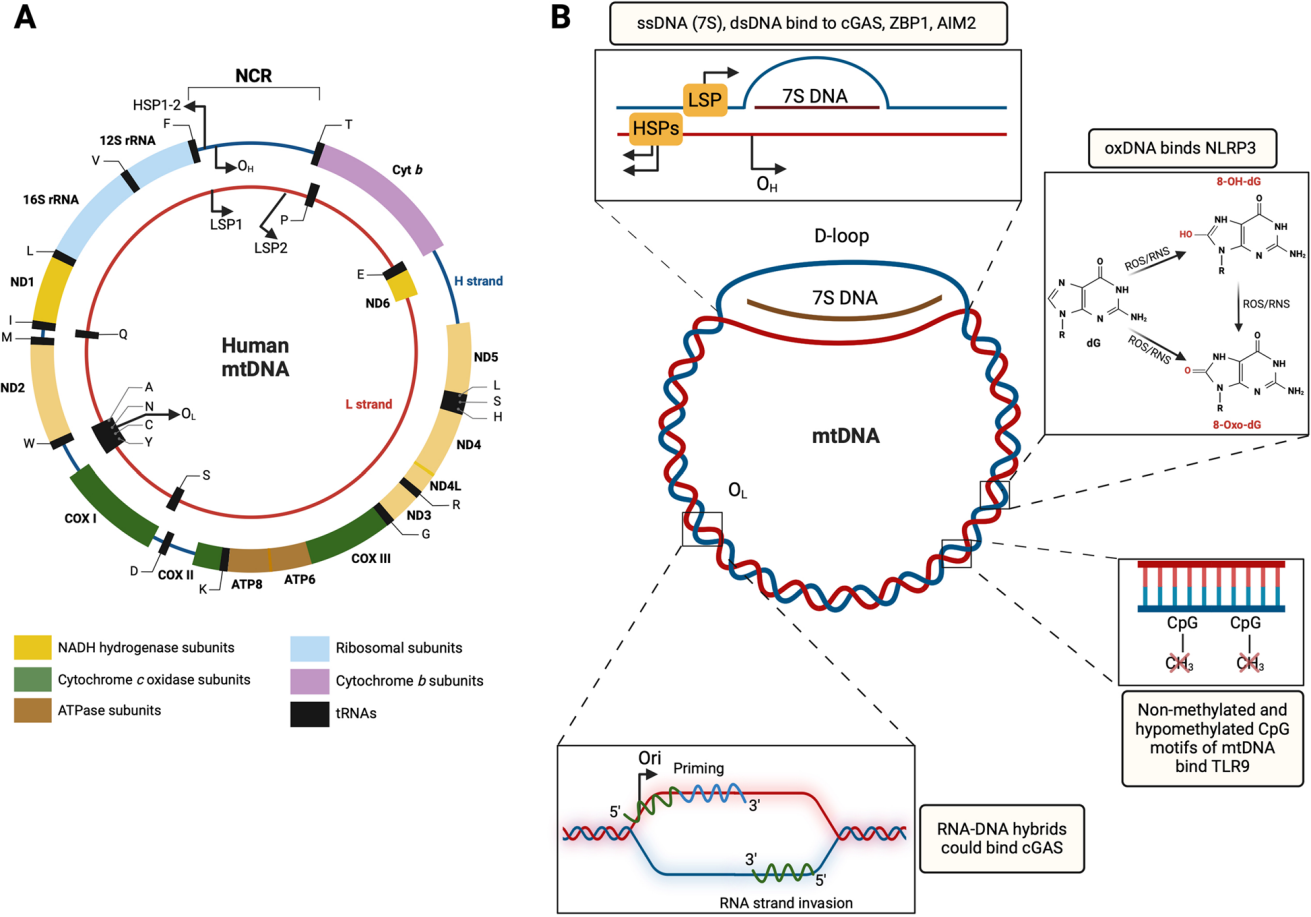
Mitochondrial genome and its immunogenic features. **A** Mitochondrial genome is a circular DNA of 16.569 base pairs, with outer heavy (H) and inner light (L) strands. It encodes for 13 proteins: NADH-dehydrogenase subunit (ND) 1, ND2, ND3, ND4, ND4L, ND5, ND6, cytochrome *b* (cyt *b*), cytochrome *c* oxidoreductase (COX) I, COXII, COXIII, ATP synthase subunit (ATPase) 6 and ATPase 8; 22 tRNAs (T, L, S, H, R, G, K, D, W, M, I, L, V, F, and P, E, S, A, N, C, Y, Q); 2 rRNA (12S, 16S). The non-coding region (NCR) includes the displacement loop (D-loop). O_H_ and O_L_ are the origins of replication on the H and L strands, respectively, whereas transcription starts from the heavy strand promoters (HSP1 and HSP2) and light strand promoters (LSP1 and LSP2). Directions of replication and transcription are indicated by arrows. **B** The mitochondrial genome harbors immunogenic features. A single-stranded DNA (7S) is formed during mtDNA replication but is not terminated, forming a three-stranded D-loop structure that could be the main source of cytosolic mtDNA. 7S may bind the cyclic guanosine-monophosphate-adenosine-monophosphate synthase (cGAS), Z-DNA binding protein 1 (ZBP1), and absent in melanoma 2 (AIM2). Deoxyguanosines (dG) of mtDNA are easily oxidized by reactive oxygen and nitrogen species (ROS and RNS) to 8-hydroxy deoxyguanosines (8-OH-dG) and 8-oxo-deoxyguanosines (8-Oxo-dG). Oxidized mtDNA binds NOD-, LRR-, and PYD- domain-containing protein 3 receptor (NLRP3) and cGAS. Mitochondrial DNA, like bacterial DNA, harbors non-methylated/hypomethylated CpG sequences that are docked by the toll-like receptor 9 (TLR9). RNA–DNA hybrids are generated during mtDNA transcription and the initial phase of the replication. Transcription starts near O_H_ (located 100 bp from LSP) by the mitochondrial RNA polymerase, generating an RNA–DNA hybrid that also primes the replication from O_H_. Similarly, RNA–DNA hybrids are generated at O_L_. During the strand invasion, RNA–DNA hybrids could potentially activate cGAS

Mitochondria are the only organelle in eukaryotes (excluding plants) that own their genome. Human mtDNA is a circular, double-stranded molecule consisting of 16,569 base pairs [17] (Fig. 1A). Compared to the nuclear genome (nDNA), the mitochondrial genome is multi-copies, highly compacted, and does not contain introns. A histone-like protein—the mitochondrial transcription factor A (TFAM) – binds mtDNA to form nucleoids, increasing its stability through compaction and controlling its expression, transmission, and degradation [17–19]. Mitochondrial DNA is rich in cytidine-phosphate-guanosine (CpG) regions that are non/hypo-methylated. The degree of mtDNA methylation, its function, and the subcellular localization of DNA methyltransferases to the mitochondria remain poorly understood, contentious, and may be cell-type specific [20–23].

The two mtDNA strands are named for their uneven distribution of guanine residues, with the heavy (H) strand having higher guanine content than the light (L) strand (Fig. 1A). Mitochondrial DNA contains only one major non-coding region (NCR), the most variable part of the genome in size and sequence [24]. The NCR includes important regulatory sequences: i) the replication origin for the H-strand (Ori_H_); ii) the L-strand (LSP) and H-strand (HSP) promoters; and iii) the displacement-loop (D-loop). The latter is a triple-stranded region that has stably incorporated a short single-stranded DNA (ssDNA) fragment known as 7S (S indicates the Svedbergs, the sedimentation unit rate in velocity gradients). Seven S is a well-known product of aborted replication events that start at Ori_H_ and stop at the termination-associated sequence [25, 26].

Human and mouse mitochondrial DNA replication and transcription require nDNA-encoded proteins, are tissue-specific, and modulated by metabolic state [27–29]. Transcription of H- and L-strands from their respective promoters results in long, intron-less, polycistronic transcripts that are processed by RNases and polyadenylated to produce 11 mRNAs, 22 tRNAs, and 2 rRNAs (12S and 16S rRNA) (Fig. 1A). The basic mechanisms of replication and transcription involve pivotal mitochondrial proteins: DNA polymerase γ (POLG), RNA polymerase (mtRNAP), TFAM, the single-stranded DNA-binding protein (mtSSB), the hexameric DNA helicase Twinkle, and the DNA topoisomerase IIIα (mtTOP3α) [17].

In addition to transcribing mtDNA, the mtRNAP generates primers for DNA replication. Replication primers originate from the light-strand promoter (LSP). However, a new light-strand promoter (LSP2) has been shown as a source of primers [30]. It has also been proposed that the mitochondrial transcription elongation factor (TEFM) interacting with mtRNAP acts as a key player in determining the switch from transcription to replication [31]. During replication, non-specific single-stranded RNA (ssRNA), double-stranded RNA (dsRNA), and RNA–DNA hybrids are generated and are usually degraded by mitochondrial RNases and DNases [32].

In the last 15 years, several studies have demonstrated that whole nucleoids and/or mtDNA fragments as ssDNA and double-stranded DNA (dsDNA) are released from the mitochondria and trigger an immune response in several pathophysiological conditions. This effect is caused by the following highly immunogenic features of mtDNA (Fig. 1B): i) it resembles bacterial DNA (double-stranded circular molecule, lacks histones, contains unmethylated CpG motifs, with the generation of aberrant DNA and RNA–DNA hybrids) [1, 2, 21, 24, 25, 33]; ii) it is much more prone to being damaged and fragmented by oxidation [34, 35]; iii) it is in multiple (hundreds to thousands) copies per cell [17], stoichiometrically favouring its binding to DNA-sensing receptors (DSRs) compared to nDNA.

Mitochondrial DNA could trigger an immune response when released: i) from the mitochondria into the cytosol and/or ii) from the mitochondria into the extracellular matrix, including the circulation. The following sections describe the main mechanisms involved in the DNA release into the cytosol and extracellular compartments.

## Mechanisms that lead mtDNA release into the cytosol

**Fig. 2:**
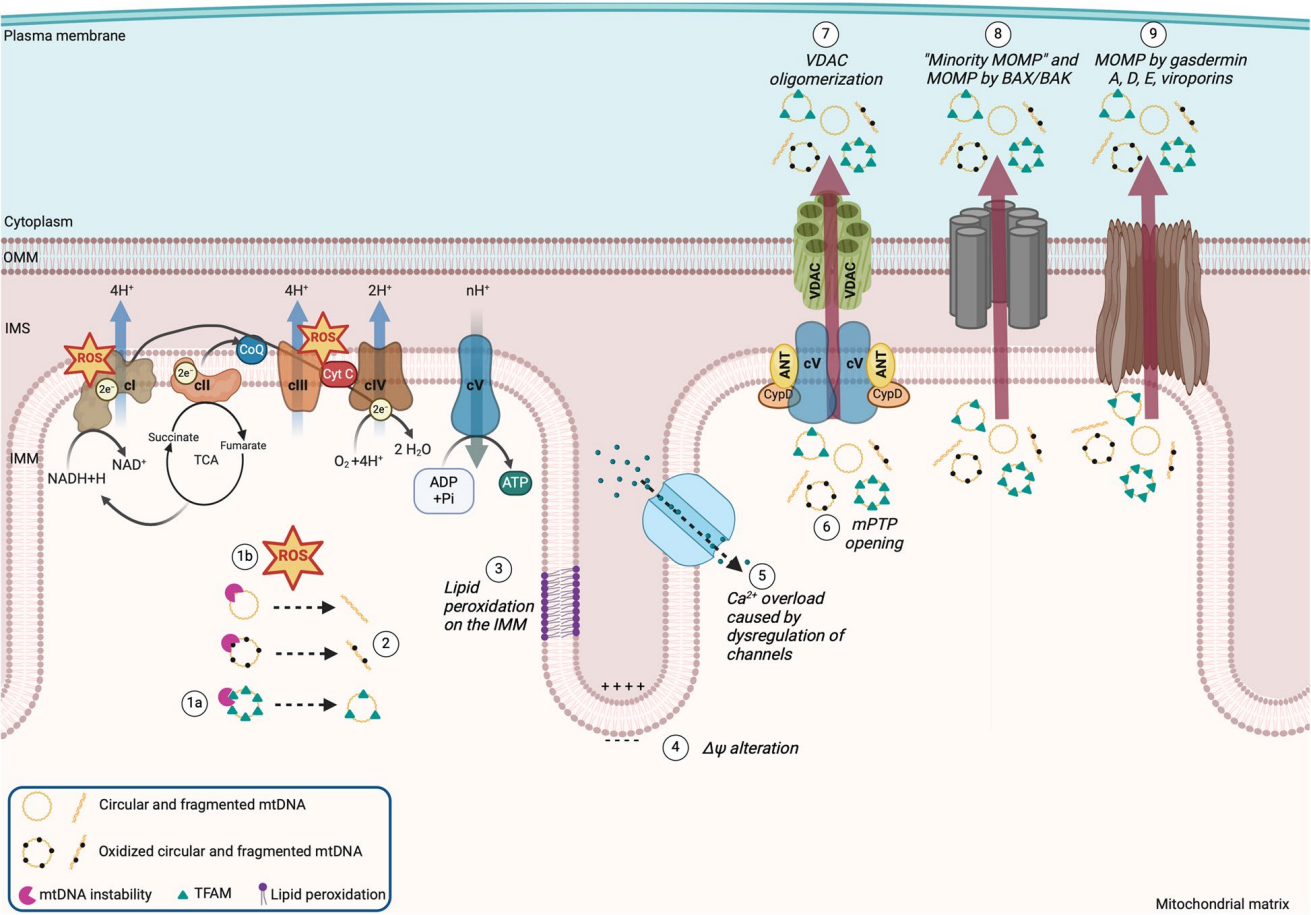
Mechanisms of mitochondrial DNA release into the cytosol. **(1a)** Loss of function of proteins involved in the mtDNA replication and transcription (like TFAM, PolG, TOP3a) causes mtDNA instability. **(1b)** An impaired electron transport system promotes electron leakage and generates superoxide anion, which is converted into additional reactive oxygen species (ROS). Mitochondrial DNA instability and ROS overproduction induce **(2)** mtDNA fragmentation, linearization, and oxidation. ROS overproduction indirectly promotes **(3)** cardiolipin and phosphatidylethanolamine oxidation (major lipid components of the IMM) and causes **(4)** alteration of the mitochondrial membrane potential (∆Ψ_mt_). Depolarization or hyperpolarization of ∆Ψ_mt_ dysregulate **(5)** mitochondrial carriers, resulting in Ca^2+^ overload in the mitochondrial matrix and consequent permeabilization of the IMM. The whole mitochondrial nucleoids and/or fragmented mtDNA are released into the cytosol by IMM permeabilization and by pores formed mainly by three different mechanisms: **(6)** mitochondrial permeability transition pore (mPTP) opening induced by transient short-lived stresses; **(7)** VDAC oligomerization. Importantly, mPTP and VDAC mainly cooperate in the extrusion of mtDNA into the cytosol. Their persistent activation could also trigger apoptosis; **(8)** oligomerization of BAX/BAK occurring meanwhile apoptotic caspases are inactive; **(9)** oligomerization of gasdermin A, or D, or E by inflammasomes. The last **(8-9)** two mechanisms lead also to the mitochondrial outer membrane permeabilization (*i.e.,* MOMP). A mechanism named **(8)** “minority MOMP” occurs when only a subgroup of mitochondria releases mtDNA by BAX/BAK oligomerization. (9) Bacteria and viruses also use specific proteins (Ply, viroporins) to create pores on the IMM and OMM, favouring mtDNA release. ^*^Of note, the exact composition of the mPTP is still debated, and how mPTP releases mtDNA is unknown. Here, we have illustrated the mPTP mainly as reported by Bonora and colleagues (59) to give the reader an indication of the complexity of the mPTP structure. Furthermore, VDAC and BAX/BAK may directly interact with mPTP (57), facilitating mtDNA by cooperative mechanisms. IMM and OMM, inner and outer mitochondrial membrane, respectively. IMS, intermembrane space; cI-V, mitochondrial complex I-V; TCA, tricarboxylic acid cycle; ∆Ψ_mt_, mitochondrial membrane potential; voltage-dependent anion channel, VDAC; ANT, adenine nucleotide translocator; CypD, cyclophilin D

**Fig. 3:**
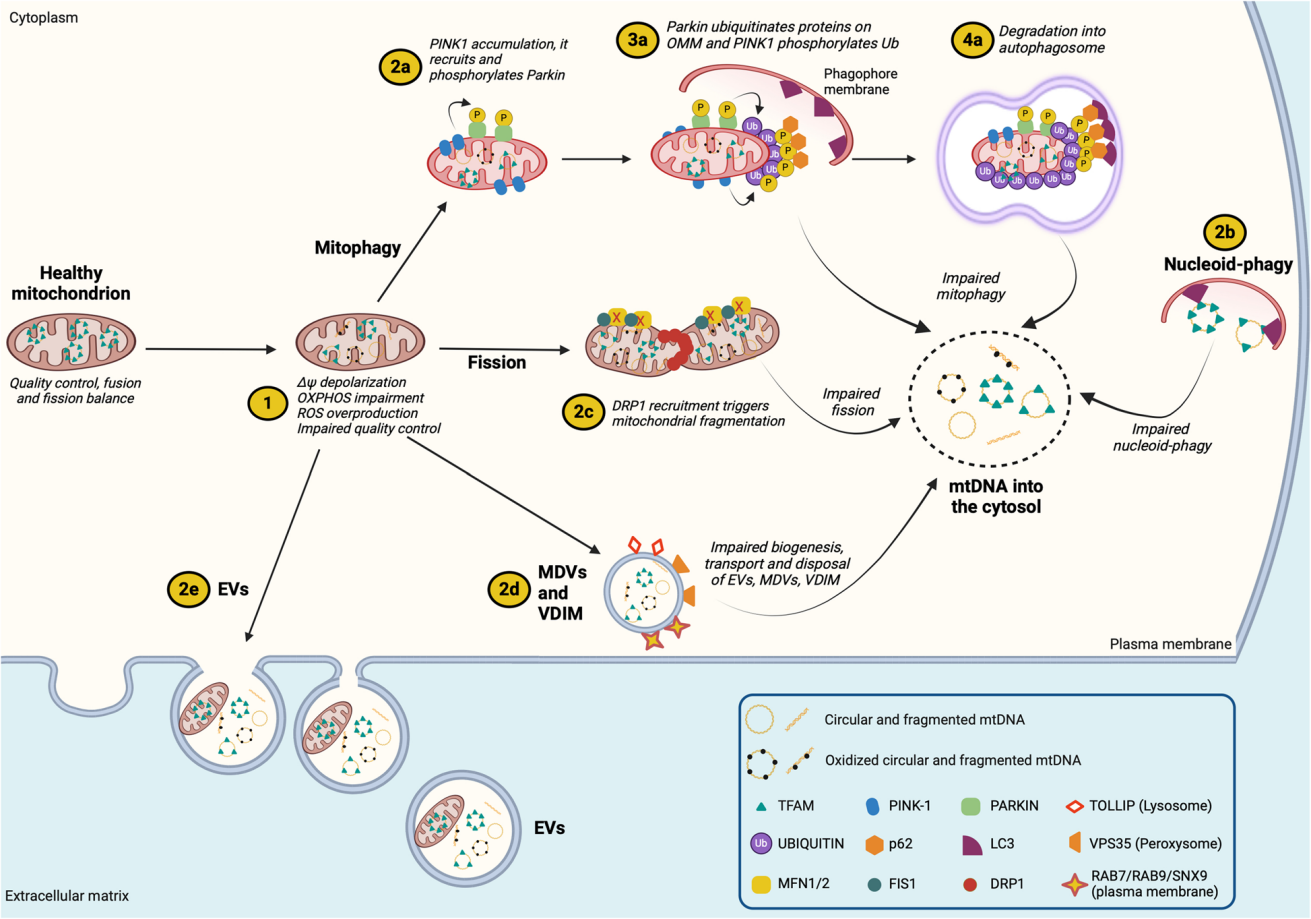
Cytosolic and extracellular release of mitochondrial DNA is caused by dysfunctional mitophagy, nucleoid-phagy, fission, and remodeling of the mitochondrial membranes. Mitochondria undergo a continuous cycle of fusion, fission, and mitophagy. Dysregulation of these processes causes mtDNA release into the cytosol and extracellularly. Mitophagy is a specific form of autophagy and a common route to remove and recycle damaged mitochondria. Two mechanisms of mitophagy are well known: non-receptor-dependent and receptor-dependent. The release of mtDNA has been described mainly by dysfunctional non-receptor-dependent mitophagy. In basal conditions, PTEN-induced kinase 1 (PINK1) is localized to the outer mitochondrial membrane as a cleaved inactive form. **(1)** Decreased ∆Ψ_mt_ prevents PINK1 cleavage, and **(2a)** promotes its accumulation, that favours **(3a)** Parkin recruitment. Parkin is an E3 ubiquitin (Ub) ligase that ubiquitinates several outer membrane proteins that are further phosphorylated by PINK1. The phospho-ubiquitinated chains (p-Ub) serve as an “eat me” signal for the recruitment of the autophagic machinery, including the adaptor protein p62 (optineurin and calcium-binding and coiled-coil domain 2, not shown). **(4a)** They interact with the microtubule-associated protein light chain (LC3), allowing the formation of a molecular bridge that encapsulates the mitochondrion in a phagophore membrane (autophagosome). Impaired or overload mitophagy causes mtDNA leaking from the autophagosome. **(2b)** Nucleoid-phagy is a form of autophagy that selectively degrades cytosolic mtDNA bound to the mitochondrial transcription factor A (TFAM). It depends on the LC3-interacting region 2 (LIR2) motif of TFAM that is recognized by LC3B, which, in turn, mediates the encapsulation and further degradation of mtDNA in the autophagosome. Impaired nucleoid-phagy allows the cytosol to retain mtDNA that has already escaped from mitochondria. **(2c)** Fission occurs on the contact site between the mitochondria and endoplasmic reticulum, and it is mainly regulated by dynamin-related protein (DRP1) and fission protein 1 (FIS1). DRP1 is recruited from the cytosol to the outer mitochondrial membrane, where it oligomerizes, forming a ring that constricts and splits the mitochondrion. FIS1 avoids mitochondrial fusion by blocking the mitochondrial fusion protein 1 and 2 (MFN1, MFN2) and optic-atrophy-1 (OPA1). Impaired fission causes the release of mtDNA into the cytosol. **(2d)** Mitochondrial-derived vesicles (MDVs, 60-150 nm), vesicles derived from the inner mitochondrial membrane (VDIM) and **(2e)** extracellular vesicles (EVs, 50-300 nm) are generated during physiological and stress conditions. The budding of the MDVs occurs at both inner and outer mitochondrial membranes and is regulated by several proteins, including the mitochondrial Rho GTPase 1 and DRP1 (not shown). MDVs are involved in intracellular quality control, allowing the degradation of irreparable proteins, lipids, and mtDNA of no yet depolarized mitochondria. The destination of the MDVs depends on the protein decoration of the outer membrane. Toll-interacting protein (TOLLIP), vacuole sorting-associated protein 35 (VSP35), Ras-associated protein 7 and 9 (RAB7/RAB9), and sorting nexin 9 (SNX9) guide the MDV toward endosomes/lysosomes, peroxisomes, and plasma membrane, respectively. VDIM have been recently reported as generated by herniation of the inner mitochondrial membrane through the oligomerization of the voltage-dependent anion channel (VDAC), and are dependent on the ROS-dependent calcium release from lysosomes. EVs are involved in intercellular communication and could transfer whole mitochondria or mitochondrial content, including mtDNA, through the extracellular matrix. The biogenesis and the regulation of the MDVs and EVs are not completely elucidated. However, impaired biogenesis and signaling to govern the destination of MDVs and EVs favours the release of mtDNA also within the cytosol

Mitochondrial DNA release into the cytosol involves single and multiple cooperative mechanisms (Fig. 2) such as i) mtDNA instability; ii) increased production of reactive oxygen species (ROS), Ca^2+^ overload, mitochondrial membrane depolarization, and mitochondrial permeability transition pore (mPTP) opening; iii) pores formed by the oligomerization of the voltage-dependent anion channel (VDAC) on the OMM; iv) pores formed by the the oligomerization of Bcl-associated X (BAX)/Bcl-2 homologous antagonist/killer (BAK); v) dysfunctional autophagy/mitophagy, remodeling of the mitochondrial membranes, and budding of mitochondrial-derived vesicles (MDVs) (Fig. 3). In the following sections, we describe these mechanisms.

### Mitochondrial DNA instability

A documented cause of mtDNA release from the mitochondria is the nucleoid instability. Several conditions are induced by the perturbation of proteins involved in mtDNA replication and transcription, such as TFAM, Twinkle, PolG, and TOP3α (Fig. 2). TFAM is a member of the high mobility group (HMG) domain protein family, and it modulates mtDNA immunogenicity by regulating mtDNA structure and abundance. TFAM structurally organizes mtDNA into the nucleoid by binding to mtDNA at every 16 bp (about 1,000 TFAM molecules for the entire mitochondrial genome) [17, 18]. TFAM is required for mtDNA replication and transcription. It is an essential gene because Tfam homozygous knockout (KO) causes embryonic lethality in mouse [36]. On the contrary, TFAM heterozygote (Het) mice are viable even with a drastic reduction of mtDNA in the heart, kidney, and liver [36]. West et al*.* (2015) showed that TFAM Het mouse embryonic fibroblasts (MEFs) displayed about 50% decreased mtDNA content and fewer, albeit larger, nucleoids compared to wild type (WT) [15]. For the first time, that study showed that the aberrant packaging of mtDNA promoted mitochondrial elongation and its release into the cytosol, where it bound the cyclic guanosine-monophosphate-adenosine-monophosphate synthase (cGAS), triggering the stimulator of interferon gene signaling (STING, Sect. "cGAS-cGAMP-STING and ZBP1-cGAS"). Mitochondrial DNA-dependent cGAS activation upregulated the expression of type I interferon (IFN-I) in TFAM Het MEFs and bone marrow-derived macrophages (BMDMs). The authors also demonstrated that the infection with herpes simplex virus 1 (HSV-1) induced TFAM downregulation, promoting mtDNA stress and IFN-I upregulation, pinpointing the biological significance of their findings. Several subsequent studies have confirmed that TFAM downregulation triggers the release of mtDNA into the cytosol and that TFAM *per se* is a modulator of immunogenicity (Sects. "Dysfunctional autophagy, mitophagy, remodeling of the mitochondrial membranes, and budding of mitochondrial-derived vesicles", "TFAM modulates mtDNA immunogenicity", "mtDNA signaling via DSRs in vascular and metabolic diseases"-"mtDNA signaling via DSRs in viral and bacterial infections") [37].

Downregulation of TFAM levels to 50% by small interfering RNA (siRNA) modifies mtDNA topology, promoting the transition from B-DNA (relaxed monomeric mt-B-DNA) to Z-DNA (catenated or supercoiled, mt-Z-DNA) [38]. Within mitochondria, two major topoisomerases are involved in the transition from mt-Z-DNA to mt-B-DNA: mitochondrial topoisomerase TOP1 (mtTOP1) and TOP3α. Non-synonymous mutated or depleted mtTOP1 led to the cytosolic release of mtDNA [39]. Recently, West’s group showed that not only TFAM Het MEFs but also cells lacking mtTOP1 or TOP3α presented mtDNA instability, nucleoid aggregation, and upregulation of the interferon stimulating genes (ISGs) in a cGAS-dependent and Z-DNA-binding protein 1 (ZBP1)-dependent fashion [40] (Sect. "cGAS-cGAMP-STING and ZBP1-cGAS").

Mutations of PolG are a common cause of mitochondrial disease, mainly affecting nervous and muscle systems [41]. The PolG mutator mouse model, expressing a proofreading-deficient 3’-5’ exonuclease, is characterized by cardiomyopathy, hearing loss, and premature aging, caused by the accumulation of mtDNA mutations, including deletion [42]. MEFs isolated from PolG mutator mice showed mtDNA in the cytosol that upregulated IFN-β by cGAS/STING [43]. PolG mutator mice also showed high levels of circulating cell-free mtDNA (ccf-mtDNA) in the plasma, probably released by macrophages, that activated innate immunity and hyperinflammation [44]. Ablation of the IFN-I-cGAS/STING signaling attenuated cardiomyopathy and extended the lifespan of the mutator mice.

The findings linking mtDNA instability and its release into the cytosol were corroborated by Sen and colleagues (2022) [45]. They showed that mtDNA damage induced by dominant negative mutations of Twinkle promoted the extrusion of nucleoids outside the mitochondria by an endosomal-mitophagy pathway (Sect. "Dysfunctional autophagy, mitophagy, remodeling of the mitochondrial membranes, and budding of mitochondrial-derived vesicles"). ATPase family AAA domain-containing 3A (ATAD3) and sorting and assembly machinery component 50 homolog (SAMM50) proteins were involved in the nucleoid extrusion, whereas the vacuolar protein sorting 35 (VPS35) mediated the maturation of early endosomes to late autophagic vesicles for the degradation. Indeed, the knockdown of Samm50 led to mtDNA release and activation of the innate immune response [45, 46].

Meiotic recombination homolog 11 (MRE11) is a nuclease that degrades nascent and damaged mtDNA when the replication fork is unstable [47]. It has been shown that MRE11 deficiency promotes mtDNA oxidation and leakage into the cytosol in T cells [48]. Paradoxically, another report showed that newly replicated mtDNA fragments processed by MRE11 activated cGAS-dependent ISGs signaling in the cytosol of SH2038 cells with defective replication fork [47].

Overall, these studies indicate that mtDNA instability caused by loss of function of the proteins involved in the replication and quality control of the mtDNA causes mtDNA leakage mainly into the cytosol.

### ROS overproduction, Ca^2+^ overload, mitochondrial membrane depolarization, and mitochondrial permeability transition pore opening

Oxidative stress (OxStr) caused by ROS overproduction is a signal for the mtDNA release from the mitochondria into the cytosol. While there are extramitochondrial sources that produce superoxide anion (O_2_^−^), such as cytochrome P450 or NADPH oxidase, O_2_^−^ is abundantly generated in the mitochondria as an inevitable consequence of ETS [49]. Electrons leaked from cI and/or cIII react with O_2_ to generate O_2_^−^, which is rapidly converted into hydrogen peroxide (H_2_O_2_) by superoxide dismutase (SOD). Hydrogen peroxide, by the Fenton reaction, oxidizes ferrous iron and forms hydroxyl radicals (OH^−^) [50, 51]. ROS (O_2_^−^, OH^−^) oxidize cardiolipin and phosphatidylethanolamine (major components of the IMM), increasing mitochondrial membrane permeability (MMP) [52, 53] **(**Fig. 2**)**. Cytosolic mtDNA is commonly detected as fragmented or oxidized because ROS reacting with the mtDNA generate 8-hydroxy-deoxyguanosine (8-OH-dG), 8-oxo-deoxyguanosine (8-oxo-dG), favouring single or double-strand breaks [35, 54, 55]. Hence, mitochondrial electron leak-generated ROS oxidize and fragment mtDNA, as well as increase MMP, favouring mtDNA expulsion. Fragments of mtDNA were detected in the cytosolic fractions of brains isolated from mice irradiated with 5 Gy (Gray, radiation unit), since ionizing radiations induce directly and indirectly (by increasing ROS) the formation of abasic sites and single-strand breaks [56]. Small (about 1 kb) fragments of mtDNA were observed after one hour of gamma irradiation, and larger fragments (about 10 kb) after 5–24 hours of irradiation. The authors concluded that OxStr mediated by ROS and induced by radiations promotes mtDNA fragmentation and increases the frequency of spontaneous opening and closure of the mPTP, contributing to the release of mtDNA fragments into the cytosol.

The signaling triggered by ROS, mitochondrial Ca^+2^, and mPTP are deeply interconnected [57]. Cytosolic Ca^+2^ concentration is low (100 nM) in physiological conditions, and it is controlled by the channels localized on the plasma membrane, endoplasmic reticulum (ER), and mitochondria. When the cytosolic Ca^+2^ level is high, Ca^+2^ is transported and accumulated within mitochondria by passive and active transports, including VDAC (Sect. "Pores formed by the oligomerization of VDAC") on the OMM and mitochondrial Ca^+2^ uniporter (MCU) channel on the IMM [58]. Ca^+2^ accumulation in the mitochondria triggers the opening of mPTP [57] (Fig. 2).

mPTP is a non-specific pore and its composition and regulatory mechanisms are not completely understood and are highly controversial [57]. Current evidence suggests that mPTP comprises a low-conductance pore, the adenine nucleotide translocator (ANT), and a full-conductance pore attributed to the cV (ATP synthase) [59] (Fig. 2). One of the most important positive regulators of mPTP opening is cyclophilin D (CypD), a chaperone located in the mitochondrial matrix that binds mPTP at the cV, in response to OxStr and pH imbalance [59, 60]. With a diameter of 1.4 nm, the mPTP allows the exchange of ions and molecules less than 1.5 kDa in size, including H_2_O, Ca^2+^, NAD^+^, and NADP^+^ [61]. Under homeostatic conditions, the mPTP is closed. Transient mPTP opening decreases ∆Ψ_mt_ and could lead to the loss of ionic homeostasis, promoting Ca^2+^ release into the cytosol and blocking mitochondrial ATP synthesis [60]. The exact mechanism by which mPTP allows mtDNA release is still unclear [62]. Considering the large dimension of mtDNA, it is unlikely that mPTP directly mediates its expulsion during the transient mPTP opening [59]. On the contrary, sustained mPTP opening causes prolonged depolarization of the ΔΨ and irreversible OMM rupture by mitochondrial swelling, promoting leakage of high copies of mtDNA into the cytosol, and activation of cell death pathways [57, 59]. The first evidence that mtDNA was released by Ca^2+^ overload and mPTP opening, was found in vitro. Isolated mitochondria (1 mg/ml) from rat liver treated with (50 nmol) Ca^2+^ showed a time-dependent decrease in ΔΨ, mPTP opening, mitochondrial swelling, and release of mtDNA into the mitochondrial buffer [63]. Release of mtDNA was inhibited by blocking mPTP opening with cyclosporin A (CsA), ruthenium red, or mitochondrial Ca^2+^-uptake inhibitors. Similarly, mPTP opening and mitochondrial swelling could be induced in isolated mitochondria from rat kidney or liver treated with (3 mM) H_2_O_2_ and (200–600 µM) Fe^2+^ in a buffer containing (50 µM) Ca^2+^. These conditions also led to mtDNA hydrolysis, followed by the release of fragmented mtDNA [64, 65].

Prohibitin 1 (PHB1) has been proposed as a regulator of mtDNA release in the IMM by mPTP opening [62]. PHB1 is a mtDNA-interacting protein located in the IMM and is indirectly involved in the maintenance of cardiolipin and phosphatidylethanolamine (PE). Macrophages and HeLa cells depleted of PHB1 showed increased release of cytosolic mtDNA by mPTP opening, which was inhibited by treatment with CsA or VBIT-4. Authors found that PHB1 maintains ∆Ψ_mt_ by inhibiting the opening of mPTP. Mechanistically, PHB1 controls mPTP formation and IMM permeability by regulating the interaction between spastic paraplegia 7 protein (SPG7) and AFG3-like protein 2 (AFG3GL2), two proteins considered core components or regulators of the mPTP (still debated). SPG7 acts like a bridge between VDAC on the OMM and PHB1 on the IMM, with PHB1 separating SPG7 and AFGL3. The authors proposed that in the absence of PHB1 in KO cells, the binding between SPG7 and AFG3L2 reinforced and favoured the opening of mPTP, exposing mtDNA to the cytosol. This was followed by mtDNA release triggering an inflammatory response [62].

Overall, these findings proved that ROS overproduction, Ca^2+^ overload, mitochondrial membrane depolarization, and mPTP opening drive and contribute to the release of mtDNA into the cytosol and are potential therapeutic targets for diseases associated with mtDNA release.

### Pores formed by the oligomerization of VDAC

The exact steps by which mPTP allows the release of mDNA through the IMM are still unknown, although sustained mPTP opening is also involved in mitochondrial swelling and rupture (Sect. "ROS overproduction, Ca2+ overload, mitochondrial membrane depolarization, and mitochondrial permeability transition pore opening"). On the contrary, VDAC – a proposed component of the mPTP [57] – plays a pivotal role in the mtDNA release through the OMM (Fig. 2) [35]. VDAC is the most abundant protein in the OMM and is encoded by three isoforms (VDAC1, VDAC2, VDAC3), with VDAC1 being highly and ubiquitously expressed [66]. VDAC has a β-barrel architecture, consisting of 19 β-strands forming a transmembrane channel, with an N-terminal containing an α-helix within the pore [67]. All three isoforms form channels, with VDAC1 and VDAC2 having similar ion selectivity and conductance, whereas VDAC3 shows different features. In addition to facilitating the passage of ions and metabolites, VDACs act as mitochondrial gatekeepers, interact with several proteins, oligomerize to form a big pore, and trigger caspase-independent apoptosis under persistent conditions of OxStr [67, 68]. Indeed, it has been shown that VDAC1 and VDAC3 are not essential for BAX/BAK-driven apoptosis in MEFs [69], whereas VDAC2 deficiency promotes apoptosis [70]. VDAC1 and VDAC3 oligomerization are triggered by mtDNA in viable MEFs harboring the deficient nuclear-encoded mitochondrial endonuclease G (EndoG) [69]. Mechanistically, mtDNA binds VDAC at its N-terminal, facilitating its oligomerization and release of mtDNA fragments into the cytosol. Of note, this event could also occur in BAX/BAK-lacking cells, indicating that the VDAC-dependent release of mtDNA through the OMM is independent of BAX-BAK permeabilization (Sect. "Pores formed by the oligomerization of BAX/BAK"). Recently, Prashar et al*.* (2024) have shown the formation of cytosolic vesicles derived from the IMM at a steady state [71]. These vesicles are devoid of OMM and are generated by the herniation of the IMM (VDIM, vesicles derived from the IMM) through the pore formed by VDAC1 oligomerization. This process occurs on the mitochondrial cristae, which contain the ETC and cV for ATP production, and require high maintenance because they are primarily damaged by OxStr. The VDIM are engulfed within lysosomes, indicating that their content is degraded by micro-autophagy. This process is enhanced by OxStr and stimulated by ROS-dependent Ca^2+^ release from the lysosomes, demonstrating that removing damaged IMM by VDIM is an intramitochondrial quality control. Interestingly, a proportion of VDMI contained ox-mtDNA, mitochondrial nucleoids, and PolG, suggesting that VDMI *per se* is a route of mtDNA release (Fig. 3) [71]. Further experiments clearly showed that VDAC oligomerization triggers mtDNA release into the cytosol in BMDMs primed with lipopolysaccharide (LPS) [35], in MEFs lacking the *i*-AAA protease YME1L (a proteolytic complex located on the IMM) [72], and in the splenocytes of a mouse model of Lupus-like disease [69]. As proof of concept, inhibition of VDAC oligomerization by VBIT-4 reduced the mtDNA release [69, 72–74]. Interestingly, proteins involved in the regulation of VDAC oligomerization indirectly affect mtDNA release. For example, hexokinase (HK), the first enzyme of the glycolytic pathway, directly binds VDAC. Recently, Baik and colleagues (2023) showed that the dissociation of HK from mitochondria triggers the VDAC oligomerization, which in turn recruits and activates the NOD-, LRR-, and PYD- domain-containing protein 3 receptor (NLRP3 inflammasome, Sects. "DNA-sensing receptors and mtDNA signaling" and "NLRP3 inflammasome") [75]. In cells depleted of mtDNA, dissociation of HK was still able to induce VDAC oligomerization but not NLRP3 inflammasome, indicating that NLRP3 aggregation to VDAC required mtDNA. Similarly, the mitochondria-associated vaccinia virus-related kinase 2 (VRK2) regulates VDAC-mediated mtDNA release, facilitating VDAC binding to mtDNA and its oligomerization [76]. The role of VDAC in mtDNA release has been recently reinforced by the findings that site-specific ubiquitination of VDAC1 by the E3 ubiquitin ligase Parkin (Sect. "Dysfunctional autophagy, mitophagy, remodeling of the mitochondrial membranes, and budding of mitochondrial-derived vesicles") prevented VDAC1 oligomerization and cytosolic release of mtDNA in hepatocytes [77]. The Parkin-dependent post-translational modification of VDAC avoided exacerbated release of mtDNA in liver fibrosis. Furthermore, it has been shown that exogenous proteins of viral origin could induce mtDNA release by modulating VDAC overexpression and oligomerization in infected cells, as recently observed for the small envelope (E) protein of SARS-Cov2 [78]. Altogether, these findings identify VDAC oligomerization as a mechanism that facilitates mtDNA release through the OMM, even without BAX/BAK activation. Given the interaction between mPTP and VDAC [57], it is plausible that mtDNA is released by a cooperative mechanism between mPTP on the IMM and VDAC on the OMM.

### Pores formed by the oligomerization of BAX/BAK

Apoptosis is an evolutionarily conserved form of regulated cell death activated in response to several stimuli, including prolonged ROS overproduction and Ca^2+^ dysregulation [79]. The mitochondrial outer membrane permeabilization (MOMP) is the point of no return of intrinsic apoptosis and leads to the release of mitochondrial proteins, including cyt *c,* into the cytosol*.* Once in the cytosol, cyt *c* binds the apoptotic protease activating factor-1 (APAF-1) to form the apoptosome, which serves as a platform for activating the caspase 9, which in turn, triggers caspase-3 and 7. Then, the cell breaks apart into several apoptotic bodies [80]. During MOMP, mtDNA is released from the mitochondrial matrix into the cytosol [81].

MOMP formation is driven by BAX and BAK, belonging to the B-cell CLL/lymphoma 2 (BCL-2) protein family. They contain nine α-helixes with the hydrophobic α5 at the protein core surrounded by the remaining amphipathic helixes. The C-terminal α9 helix contains a transmembrane domain that anchors the proteins to the OMM. BAX and BAK undergo conformational changes upon activation that facilitate hetero- and homo- oligomerization. The BAX/BAK oligomer resembles an amphipathic polypeptide and destabilizes the lamellar structure of the OMM, forming pores [82] (Fig. 2). Using MEFs, White et al. (2014) demonstrated for the first time that BAX/BAK pores triggered mtDNA release into the cytosol, promoting the upregulation of IFN-β by cGAS [83] (Sect. "cGAS-cGAMP-STING and ZBP1-cGAS"). Interestingly, the authors showed that caspase-3, -7, or -9 activation blocked mtDNA signaling. Rongvaux et al*.* (2014) described a similar mechanism where, in the absence of active caspases, MOMP by BAX/BAK induced mtDNA release and cGAS-dependent IFN-I response [14]. Confocal and lattice light-sheet microscopy revealed that BAX/BAK oligomerization in MEFs caused the MOMP-associated efflux of cyt* c* and mtDNA release. MOMP formation was accompanied by alteration of mitochondrial morphology, with herniation of the IMM, and a consequent mtDNA leakage to the cytosol that did not involve mPTP opening [84]. Riley from Tait’s group (2018) found very similar results and described how BAX-formed pores are dynamic, they grow over time and allow for the extrusion of the IMM, by which mtDNA is released after permeabilization [85]. However, both studies did not clearly explain how the herniated IMM could be permeabilized or lose integrity to allow mtDNA to be released into the cytosol. There are indications of BAX/BAK contributing to the formation of mPTP channel, facilitating the interaction between IMM and OMM, and in a way, sensitizing mPTP [57]. In particular conditions, it may be possible that BAX/BAK modulate mPTP or even promote permeabilization of the IMM. Importantly, MOMP, mitochondrial herniation, and mtDNA release with consequent upregulation of IFNs by cGAS signaling was prevented only when both BAX and BAK genes were deleted by CRISPR-Cas9, in caspase-inhibited conditions. The specific deletion of BAX or BAK was insufficient to prevent MOMP and mtDNA release [85]. These results were confirmed by Cosentino et al*.* (2022), which also nicely explain the unique features of BAK and BAK and their interplay to form pores [86]. They showed that i) although BAX and BAK exhibit high homology in sequence and structure, at the steady-state, the BAX inactive forms are mainly localized into the cytosol, whereas BAK inactive forms are predominantly located at the OMM; ii) functionally, in the OMM of the apoptotic cells, BAK assembles in lines, arcs, and rings that are small and narrowly distributed, whereas BAX assembles in bigger and dispersed distributed structures; iii) during the oligomerization, BAK oligomerizes faster than BAX, whereas BAX slowly accumulates and enlarges the growing pore; iv) although BAK and BAX have different oligomerization properties during apoptosis, they regulate each other and co-assemble into supra-molecular structures; v) BAX and BAK reciprocal regulation controls the growth of the pores, the kinetics of the release of the mitochondrial content into the cytosol, including mtDNA, and indirectly, the activation of the cGAS-STING pathway in cells treated with pan-caspase inhibitors [86]. In summary, BAK and BAK assembly rate regulated the apoptotic pore's growth size, with BAK oligomerizing faster and accelerating mtDNA release into the cytosol than BAX [86]. As large BAX/BAK pores are formed, mtDNA entering into the cytosol can no longer continue to drive the synthesis of IFNs by cGAS signaling because the cell succumbs to apoptosis driven by caspases [81]. Of note, it has been recently shown that BAX pore activity and the consequent cGAS-STING activation are dependent on the enrichment of polyunsaturated lipids in the membrane, with the fatty acid desaturase 2 (FADS2) playing a pivotal role [87]. Summing up all the findings, due to the order of events, apoptotic cells release mtDNA into the cytosol only during the early apoptotic phase [81] or in conditions in which caspases are impaired, dampened, or inhibited [85, 86].

Consistent with the “early apoptosis events” model, it has been recently shown that sublethal apoptotic stress in senescent cells triggers mtDNA release into the cytosol by minority MOMP (miMOMP) [88], a phenomenon in which a subset of mitochondria undergo MOMP in a stress-regulated manner [89]. Interestingly, authors showed that: i) miMOMP was caused by BAX oligomerization occurring in a subset of mitochondria in senescent but not proliferating fibroblasts; ii) miMOMP promoted the release of mitochondrial nucleoids that engaged cGAS-STING to upregulate senescence-associated secretory phenotype (SASP) genes. As proof of concept, human senescent fibroblasts depleted of mtDNA failed to upregulate SASP genes, whereas transfection with mtDNA restored SASP expression*;* iii) SASP expression was significantly decreased in the liver of mice lacking BAX/BAX, previously irradiated with 4 Gy for six days or aged for 20 months; iv) treatment for three months of aged mice with BAI1—a small molecule preventing BAX translocation and oligomerization—ameliorated age-related decline of neuromuscular coordination, bone microarchitecture, and delayed frailty symptoms; v) BAI1-treated mice showed reduced expression of SASP in the bone and the whole brain, indicating that blocking mtDNA release by inhibiting miMOMP improved the healthspan. Altogether, these findings finely dissect the mechanism by which mtDNA is released during MOMP and miMOMP caused by BAX/BAK oligomerization.

In this context, it needs to be highlighted that because intrinsic apoptosis is a conserved process in metazoan, evolutionary linked to the endosymbiotic event (Sect. "Mitochondrial origin and functions"), and naturally occurring during the developmental process and homeostatic cell turnover within tissues [81, 90, 91], caspase activation during the apoptotic process driven by BAX/BAK oligomerization aims to control cell clearance and avoid the immune response. Considering the presence of multiple mechanisms of mtDNA release in living cells, in the absence of inflammasome activation (Sect. "AIM2 inflammasome"-"Priming and activation of the AIM2- and NLRP3-dependent inflammasome"), it is plausible that permeabilization of the OMM by pores depends on the level of mitochondrial stress, with VDAC oligomerization mainly triggered during moderate stress, while BAX/BAK could be activated during sublethal and lethal stress that leads to caspase-independent apoptosis.

### Dysfunctional autophagy, mitophagy, remodeling of the mitochondrial membranes, and budding of mitochondrial-derived vesicles

Autophagy is a degradative process that removes misfolded or aggregated proteins and damaged or redundant organelles [92]. It is stimulated by cell starvation, aging, specific mutations, and exposure to xenobiotics. Mechanistically, damaged proteins or organelles are targeted and engulfed in a double-membraned vacuole (autophagosome), which fuses with a lysosome (autophagosome-lysosome), allowing the cargo to be degraded by hydrolases and proteases. The recycled materials are released in the cytoplasm by lysosomal permeases.

Accumulation of dysfunctional mitochondria that could not be degraded by autophagy favours mtDNA release into the cytosol. As evidence, the depletion of specific autophagic proteins such as microtubule-associated protein 1 light chain 3 β (LC3B) or beclin-1 led to the release of mtDNA into the cytosol in macrophages activated with LPS [93]. The inefficient autophagic flux activated caspase-1 and consequent release of IL-1β and IL-18. Similarly, the impasse of the autophagy-mediated degradation of mtDNA in cardiomyocytes of DNase II-deficient mice (lysosomal DNase) increased the accumulation of cytosolic mtDNA, causing inflammation and hypertrophic heart failure [94]. Confirming the link between mtDNA release and autophagy, other authors showed that autophagy is essential in cleaning cytosolic mtDNA induced by irradiation in cancer cells, and it blunted the abscopal response triggered by IFN-I expression cGAS-dependent [95].

Mitophagy is the selective degradation of damaged mitochondria, and its initiation involves proteins regulating mitochondrial fusion, fission, and quality control [96]. Low ∆Ψ_mt_ activates PTEN-induced kinase 1 (PINK1) that recruits Parkin by phosphorylating Ser65, which in turn ubiquitinates mitofusin-1 and -2 (MFN1, MFN2), VDAC, translocase of the outer membrane (TOM), fission 1 (FIS1), to stimulate the sequestration of mitochondrion in a autophagosome, that further fuses with the lysosome [97, 98]. The deficiency of proteins involved in the mitophagy causes mtDNA release (Fig. 3). PINK1 and Parkin prevent inflammation by removing damaged mitochondria, as evidenced by *Pink1* and *Parkin* KO mice showing reduced mitophagy but increased serum ccf-mtDNA [99]. Similarly, PINK1 deficiency induced mtDNA release in type II alveolar epithelial cells (AECII) in a model of idiopathic pulmonary fibrosis (IPF) [100]. Reciprocally, mitophagy activation by urothelin A treatment decreased mtDNA release and cGAS-dependent inflammation in the retina of aged mice and ARPE-19 cell line [101]. Similar results were found in a mouse model of autosomal dominant tubulointerstitial kidney disease due to uromodulin mutations (ADTKD-UMOD), in which cGAS was activated by cytosolic mtDNA [102]. Boosting mitophagy mitigates STING activation and attenuates tubular injury.

A new insight into the connection between mtDNA and its disposal has been recently brought by Shadel’s group [103]. Newly replicated nucleoids usually segregate by polymerization of the ER-associated actin, followed by mitochondrial fission. Cells depleted of TFAM, TOP3α, or with mtDNA replication defects caused by HSV-1 UL12.5 protein (alkaline DNase, targeting mtDNA) exhibited elongated mitochondria and enlarged nucleoids, with stalled ER-actin polymerization and mitochondrial fission. These replication-incompetent nucleoids colocalized with early and late endosomal markers (RAB5 and RAB7, respectively), indicating that they are trafficked to the endosomes for their disposal [103]. Interestingly, endosomal rupture enabled cGAS to access mtDNA, suggesting that the inefficient disposal of replication-incompetent nucleoids could also favour the escape of the nucleoids from the endosomes to the cytosol (Fig. 3).

A protein involved in the mitochondrial fission is the dynamin-related protein 1 (DRP1). DRP1 promotes mitochondrial fragmentation and further degradation of fragments in autophagosomes [104] (Fig. 3). Upregulation of DRP1 promoted autophagosome engulfment, increased mtDNA in the cytosol, and triggered chemokine secretion in hepatocellular carcinoma [105] and Kupffer cells treated with LPS [106]. A screen performed by CRISPR KO targeting mitochondrial regulators for the IFN-I response showed that an intact cristae architecture maintained by several proteins involved in the mitochondrial fission, fusion, and mitophagy prevents mtDNA release and the activation of the cGAS-STING-dependent inflammation [107]. These results were corroborated by Irazoki et al*.* (2023), which showed how opposite mitochondrial morphologies drive distinct inflammatory pathways [108]. The data indicate that *Fis1* or *Drp1* KO myoblasts displayed mitochondrial elongation and mtDNA release in the cytosol, which activated mainly cGAS. On the contrary, *Mfn1* and *Mfn2* KO myoblasts showed fragmented mitochondria with endosomal mtDNA that activated the toll-like receptor-9 (TLR9).

A new mechanism links metabolism with mitochondrial membrane remodeling and release of mtDNA into the cytosol through MDVs [109]. MDVs are small vesicles (60–150 nm diameter, 0.15 µm^2^ area) generated from mitochondria, with an autonomous membrane potential, carrying mitochondrial content to communicate with other organelles [110, 111] (Fig. 3). Typical protein markers of MDVs are the translocase of the outer mitochondrial membrane (TOM20), the mitochondrial-anchored protein ligase (MAPL), and the pyruvate dehydrogenase E2/E3-binding protein (PDH). Frezza’s group recently showed that the accumulation of fumarate induced the formation of swollen-elongated mitochondria that release mtDNA into the cytosol with consequent upregulation of the ISG expression mediated by the cGAS-STING pathway [109]. The authors reported that the mtDNA release relies on the function of the sorting nexin 9 (SNX9), an endocytic accessory protein controlling the formation/destination of the MDVs. Silencing SNX9 arrested the formation of MDVs at the mitochondrial membrane, preserved membrane integrity, and decreased the release of mtDNA and the activation of the STING pathway. These results highlight how the accumulation of a TCA metabolite like fumarate indirectly controls the SNX-MDV-dependent release of mtDNA and modulates innate immunity. Altogether, these findings point out that dysfunctional proteins involved in mitophagy, mitochondrial dynamics, membrane remodeling, and the formation of MDVs lead to mtDNA release into the cytosol.

## Mechanisms that lead mtDNA release into extracellular environments

**Fig. 4:**
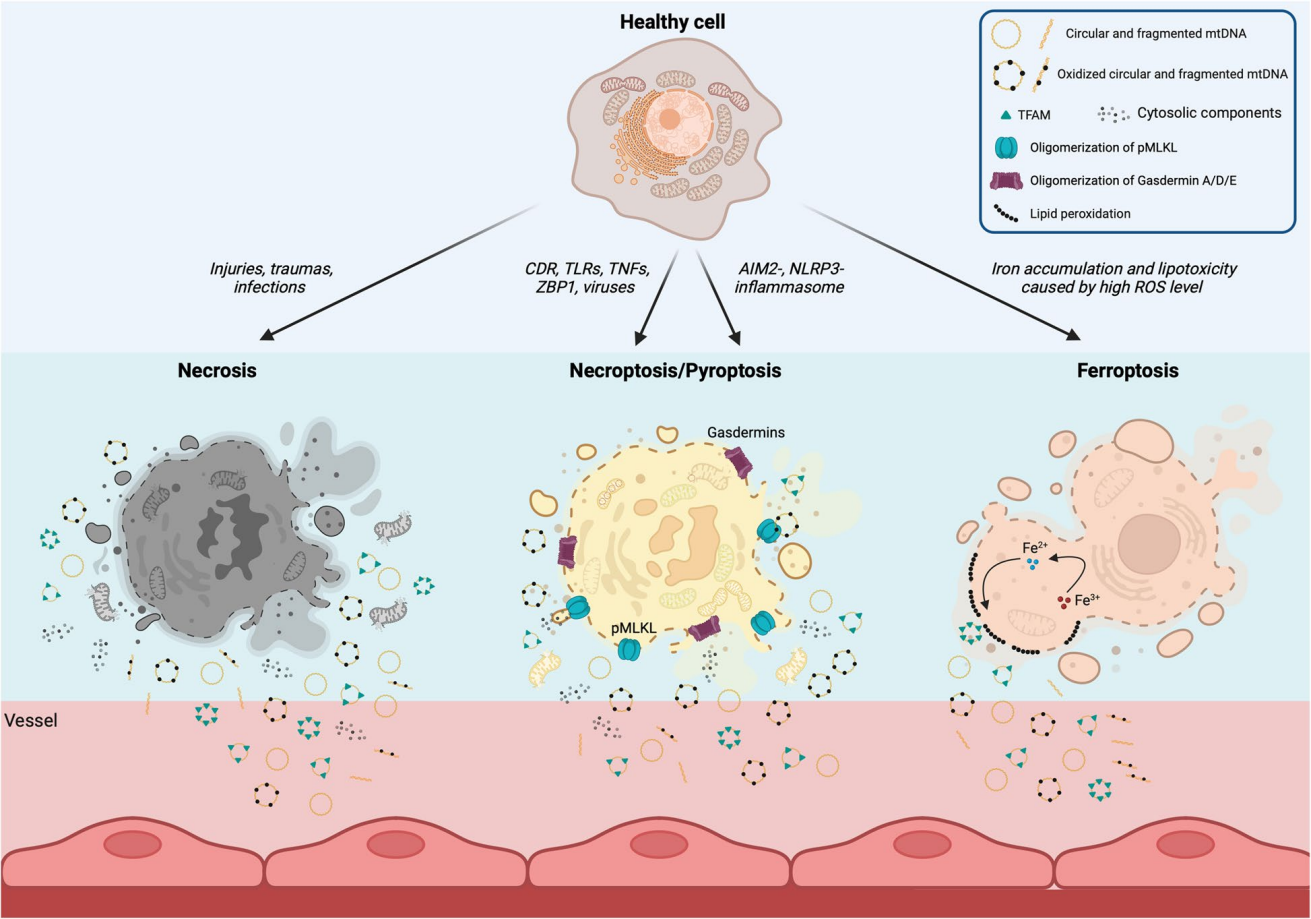
Mechanisms of mitochondrial DNA release in the extracellular environments by cell death. Mitochondrial DNA is released in the extracellular matrix, including serum, mainly by the following cell deaths: necrosis, necroptosis, pyroptosis, and ferroptosis. These mechanisms of cell death have the same outcome: the permeabilization of the plasma membrane (PM) and its rupture by which mtDNA is released. Necrosis is an uncontrolled cell death induced by several injuries, traumas, and infections that suddenly cause loss of membrane integrity, allowing the discharge of intracellular content, including mtDNA. Necroptosis is initiated by ligands activating cell death (CDR), specific toll-like receptors (TLR) like FAS, tumor necrosis factors (TNFs), ligands for Z-DNA binding protein (ZBP1), and viral infections. The necroptotic cascade terminates with the oligomerization of phosphorylated mixed lineage kinase domain-like protein (pMLKL) that forms pores on the PM. Pyroptosis is similar to necroptosis, but its cascade signaling is triggered by the AIM2- or NLRP3- inflammasomes, that are activated by pathogen- and damage-associated molecular patterns (PAMPs and DAMPs). The signaling leads to the primary permeabilization of the PM by oligomerization of A, D or E gasdermins with consequent release of IL-1b and IL-18. The complete permeabilization of the PM also allows the release of mtDNA. Ferroptosis is a regulated cell death caused by iron overload that induces lipid peroxidation by the Fenton reaction (H_2_O_2_+Fe^2+^) and lipoxygenases. It is triggered by reactive oxygen species (ROS) not well neutralized by antioxidant defenses (decreased intracellular glutathione and activity of glutathione peroxidase). Lipid peroxidation permeabilizes the mitochondrial and plasma membranes, allowing the formation of pores and micelles by which cytosolic components and mtDNA leaking from the mitochondria are discharged

The most relevant processes that cause extracellular mtDNA release are specific types of cell death (necrosis, necroptosis, ferroptosis, pyroptosis) (Fig. 4) and the coupling of dysfunctional autophagy (described in the previous section) with exocytosis. Additionally, studies have provided evidence of respiratory-competent mitochondria in the plasma [112], suggesting that mtDNA can also be released into circulation upon lysis of whole circulating mitochondria.

Necrosis is a premature cell death induced by chemical and physical insults that promote plasma membrane (PM) rupture, causing extracellular mtDNA release. Necrosis is driven by prolonged mPTP opening, mitochondrial swelling, and release of apoptogenic factors (including cyt *c*). During necrosis, caspases and apoptosomes are not activated, usually due to insufficient levels of ATP [113]. Necrotic tissues induced by trauma released mtDNA and other damage-associated molecular pattern (DAMP) into the circulation [114]. High plasma levels of ccf-mtDNA were detected in COVID-19 patients who required admission to the intensive care unit (ICU), were intubated and/or died [115]. Interestingly, ccf-mtDNA levels positively correlated with IL-6 and lactate dehydrogenase levels, well-known markers of necrosis, suggesting that SARS-CoV2-infected necrotic cells release mtDNA. Bliksøen et al*.* (2012, 2016) showed that human myocardial necrosis caused by infarction increased ccf-mtDNA levels and that ccf-mtDNA was endocytosed in cardiomyocytes, activating TLR9 [116, 117]. Similar results were detected in a model of cardiac injury in which necrosis was caused by ischemia/reperfusion (I/R) [118].

Necroptosis is a caspase-independent form of cell death characterized by controlled cell membrane lysis that facilitates the release of mtDNA into the extracellular space. This process is driven by the phosphorylation of the mixed lineage kinase domain-like (MLKL) protein, which promotes its oligomerization, forming pores on the PM. The receptor-interacting serine/threonine protein kinases 1 and 3 (RIPK1 and RIPK3) are the main players in the activation of MLKL [119]. Mangalmurti’s group determined the distribution of two forms of mtDNA, ccf-mtDNA and the mtDNA bound to the red blood cells (RBCs) in human and mouse plasma under normal conditions or following necroptotic cell death. Under basal conditions, they found that most of the CpG-DNA was bound to RBCs by TLR9, whereas traumas and systemic inflammation increased the fraction of unbound mtDNA. Loss of CpG-DNA sequestration by RBCs exacerbated lung injury, suggesting that RBCs scavenge ccf-mtDNA to alleviate lung inflammation [120]. Interestingly, *Ripk3* KO mice showed less RBC-bound mtDNA than WT mice following necroptosis induction, suggesting that preventing RIPK3-mediated necroptosis blunts mtDNA release. Zhang and colleagues (2020) also observed that critically ill patients with intra-abdominal infection had correlating high levels of circulating RIPK3 and ccf-mtDNA [121]. Interestingly, mtDNA was released into the cytosol in a mammary tumor-derived cell line under glucose deprivation [122]. Released mtDNA bound to the ZBP1 and triggered necroptosis by MLKL. Altogether, these studies provide compelling evidence that mtDNA release can result from and lead to necroptosis.

Pyroptosis is a lytic cell death induced by caspase-1 and -3 activation. Caspase-1 triggers the formation of pores on the plasma and mitochondrial membranes by the N-terminus of gasdermin D (GSDMD) [123] (Figs. 2, 4). Similarly, caspase-3 promotes pore formation by cleaving gasdermin A and gasdermin E [124, 125]. Permeabilization of PM by gasdermins induces a rapid MOMP-driven mitochondrial collapse and the accumulation of mtDNA in the cytosol that facilitates the extracellular release of mtDNA by PM rupture [126]. Recent data suggested that ox-mtDNA interacts with GSDMD and stabilizes its oligomerization in neutrophils, generating a vicious cycle that further promotes extracellular mtDNA in systemic lupus erythematosus (SLE) [127].

Ferroptosis is a necrotic cell death caused by excessive iron-dependent lipid peroxidation. Cells undergoing ferroptosis display decreased mitochondrial volume and damage/rupture of the OMM and PM, which facilitate mtDNA release [119, 128]. Iron accumulation is also known to cause mtDNA breaks and decreased mtDNA transcription [129]. Li and colleagues (2020) showed that the nucleoside analog 2ʹ, 3ʹ-dideoxycytidine (ddC, Zalcitabine, an antiviral drug) induces ferroptosis and mtDNA release [130]. Mechanistically, ddC is a replication chain terminator that leads to mtDNA replication failure, increasing linear mtDNA fragments [55]. Exposure to ddC promoted TFAM degradation, decreased mtDNA copy number, oxygen consumption, ATP production, and increased ROS, with cytosolic release of ox-mtDNA activating cGAS-STING mediated-autophagy [130]. In this case, cytosolic mtDNA was the triggering signal inducing cell death by ferroptosis, with a plausible extracellular release of the cytoplasmic content, including mtDNA.

Exocytosis caused by dysfunctional mitochondrial transport, autophagy, and mitophagy could induce extracellular mtDNA release. For instance, mutations of Desmin, a central intermediate filament required for correct positioning and function of several organelles, including mitochondria, decreased mitochondrial respiration, ∆Ψ_mt_, ATP/ADP ratio, and induced extracellular mtDNA release [131]. As proof of concept, treatment with GW 4869, an exocytosis inhibitor, reduced the amount of extracellular mtDNA, emphasizing the role of exocytosis in mtDNA release. Release of EVs enriched of mtDNA were found in the conditioned medium of BEAS-2B cells exposed to cigarette smoke extract [132] and in an in vitro model of Huntington’s disease (fibroblasts and neural stem cells) [133] **(**Fig. 3**)**. Similarly, autophagy-induced release of mitochondrial contents, including mtDNA, has been observed in rat hepatocytes and MEFs treated with LPS [134]. In these cases, mtDNA secretion was mediated by the exocytosis of autolysosomes and could be inhibited upon treatment with 3-methyladenine (3MA), an autophagy inhibitor. Nicolás-Ávila et al. (2020) also demonstrated that during cardiac stress, impaired autophagy led to the extrusion of defective mitochondria containing mtDNA by exophers, which were then taken up and destroyed by macrophages [135]. Impairing this process of mitochondrial clearance determined ventricular dysfunction. These studies highlight that cell deaths, dysfunctional autophagy and exocytosis, and impaired mitochondrial transport are relevant sources of mtDNA release in the extracellular matrix, including blood.

### Mitochondrial DNA in the extracellular traps

Extracellular mtDNA plays a key role in the formation of the extracellular traps (ETs), a response of the immune system mainly induced by bacterial, viral, and parasite infection [136, 137], but also by traumas [138], cancer [139, 140], and autoimmunity [141–143]. ETs consist of extracellular filaments of decondensed chromatin (mtDNA and/or nDNA), citrullinated histones, microbicide proteins like elastase, myeloperoxidase, and defensins. The function of ETs is to trap and kill bacteria, viruses, and parasites in the extracellular matrix [144]. However, prolonged and excessive production of ETs becomes harmful to the tissues [136, 145]. ETs are generated by cytolysis and/or by specific secretion of granules (degranulation) of neutrophils (NETs), eosinophils (EETs), or basophils (BETs) [136]. Yousefi and colleagues demonstrated that mtDNA is released from viable neutrophils to form NETs [146], from eosinophils to form EETs [147], and from basophil to form BETs [148]. In this context, mitochondrial-dependent [146, 149, 150] and independent ROS production [151, 152] triggers specific degranulation or a unique form of programmed cell death of neutrophils (NETosis) and eosinophils (EETosis) [137, 144, 153].

Increased ROS caused by mitochondrial dysfunction contributes to sickle cell disease (SCD) [154, 155]. Patients with SCD showed abnormal retention of mitochondria in mature RBCs and higher levels of ccf-mtDNA in the plasma compared to healthy controls, with ccf-mtDNA from the plasma of SCD patients triggering the formation of NETs in vitro [156]. Similarly, neutrophils isolated from patients affected by chronic obstructive pulmonary disease (COPD) upon exposure to cigarette smoke (CS) showed NETosis that depends on mitochondrial ROS (mtROS) [150]. Extracellular mtDNA driving the formation of NETs was also reported in the alveolar compartment during lung ischemia–reperfusion injury [157] and in patients with bone fractures after injury and post-orthopedic trauma surgery [138]. Multiple and amplified mtDNA signals leading to IFN-I production have been shown in SLE (an autoimmune disease characterized by the production of antibodies that react against self-antigen), including activation of cGAS by RBCs retaining mitochondria, unconventional production of IL-1β by monocytes [158] and NETs enriched of ox-mtDNA [142]. The levels of antibodies against ox-mtDNA were elevated in the serum and positively correlated with disease severity in SLE patients, which also showed abundant mtDNA deposited in the NETs of their renal biopsies [141]. Caielli et al*.* (2016) confirmed these findings and reported that activated SLE neutrophils are not able to disassemble ox-mtDNA from TFAM, a process that is essential for the disposal of ox-mtDNA into lysosomes, since neutrophils are constitutively unable to complete mitophagy upon mitochondrial damage [143]. The lack of disposal leads to the accumulation of oxidized mitochondrial nucleoids (ox-mtDNA bound to TFAM) within the neutrophils, which extrude them without cell death and membrane disruption. Another study by the same group, showed impaired mitophagy in the RBCs of the SLE patients during erythroid cell maturation, with release of mtDNA that triggers the production of IFN by activating cGAS-STING in macrophages [159]. Finally, independent studies have proved that mDNA release is one of the major driver of SLE: i) blocking VDAC oligomerization to decrease mtDNA release ameliorates the symptoms of SLE in a mouse model [69]; ii) decreased ROS production by metformin treatment (inhibitor of complex I and NADPH oxidase activities, involved in the NET formation by mtDNA release) reduced the disease flares in patients with mild and moderate SLE [141, 160]. Increased ROS production, mitochondrial Ca^2+^ overload, and reduced ∆Ψ_mt_ caused the release of ox-mtDNA and NETs also in the Lupus-like disease model [142]. Similarly, it has been recently shown that ox-mtDNA is released by pyroptotic platelets in a mitochondrial ROS-dependent fashion, exacerbating NETs formation in a model of sepsis [161].

Eosinophils are essential immune cells that respond to allergic disorders and helminthic parasitosis [136]. Yousefi et al*.* (2008) reported for the first time, that viable eosinophils triggered with LPS release mtDNA to create a scaffold that secures a high concentration of granule proteins, forming ETs to curb and kill bacteria [147]. Using a mouse model of sepsis caused by cecal ligation and puncture, they showed that eosinophil infiltration and mtDNA deposition have a functional antimicrobial role in vivo*.* Another study links the release of the EETs to the thymic stromal lymphopoietin (TSLP), a cytokine that is expressed in the epithelial cells of the intestine, airways, and skin, and is upregulated in bronchial asthma, dermatitis, and allergy [162]. It has been shown that the TSLP receptor is localized on the eosinophils of the blood and infiltrated in the biopsies of skin isolated from patients, but absent in neutrophils. Activation of the receptor by TSLP released during the disruption of the epithelial barrier, directly stimulates eosinophil to release EETs enriched with mtDNA and eosinophilic cationic protein in a concentration-dependent manner, inhibiting the growth of commensal bacteria that could invade damaged skin [162]. Of note, the formation and composition of EETs are still highly debated [136]: some studies showed that EETs are formed by a specific cell death named EETosis rather than released by viable cells. Other studies indicate nDNA as the major component of the EETs compared to mtDNA, suggesting the presence of multiple situations.

Basophils are involved in the late phase of the pro-inflammatory response to allergenic and parasitic stimuli, and produce extracellular traps that contain mtDNA, but not nDNA [148]. Indeed, human basophils primed with IL-3 and stimulated through the IgE receptor, release mtDNA in a ROS-dependent but cell death-independent manner. Interestingly, blocking mtROS by MitoQ abrogated the release of mtDNA, indicating that mtROS are required for BETs. The presence of BETs was validated in biopsies of skin isolated from patients with bullous pemphigoid, eosinophilic folliculitis, and Welll’s syndrome, and in a mouse model with basophil infiltration [148]. In a second study, the same group reveals the ability of the basophils to kill bacteria through BETs [163]. Altogether, these findings identified extracellular mtDNA as a structural and functional component of the NETs, EETs, and BETs.

## Mechanisms of mtDNA release caused by bacterial and viral infections

Bacterial and viral infections have been shown to trigger mtDNA release into the cytosol and extracellularly by unique and overlapping mechanisms. Some of them, like ROS overproduction, Ca^2+^ overload, and pore formation, were previously described (Sect. "ROS overproduction, Ca2+ overload, mitochondrial membrane depolarization, and mitochondrial permeability transition pore opening"). For example, *Streptococcus pneumoniae (Sp),* a Gram-positive bacterium, promotes mtDNA release in two ways. First, pneumolysin (Ply, its major virulence factor) forms pores (250–350 Å) on the PM of alveolar epithelial cells, causing Ca^2+^ influx and inducing mPTP opening with the release of mtDNA into the cytosol and circulation [164, 165]. Second, *Sp* secretes high levels of H_2_O_2_ that causes mitochondrial damage and oxidizes mtDNA, indirectly fostering mtDNA release [166]. Another trigger of mtDNA release is LPS, a major component of the outer membrane in the Gram-negative bacteria. Huang et al*.* (2020) demonstrated that LPS activated GSDMD by caspase-1, which permeabilizes directly the mitochondrial membranes, leading to mtDNA into the cytosol [167]. Viruses also force mtDNA release into the cytosol and extracellularly. Many viruses encode for viroporins, small hydrophobic proteins that oligomerize on the membrane of host cells and lead to the formation of hydrophilic pores. For instance, influenza and encephalomyocarditis viruses use viroporins to permeabilize mitochondria [168, 169]. Other mechanisms used by the viruses include Ca^2^ ^+^ influx, mPTP opening, decreased ∆Ψ_mt_ [76, 170–172], and cell lysis (Sect. "Mechanisms that lead mtDNA release into extracellular environments"). Altogether, these studies indicate that bacteria and viruses have multiple mechanisms to hack mammalian cells, promoting intracellular and extracellular mtDNA release.

## The first response to DNA mislocalization: exonucleases and endonucleases

Cells have developed a specialized response to mislocalized DNA to prevent the activation of immune responses. Deoxyribonucleases (DNases) recognize DNA and cleave the phosphodiester bond between nucleotides. They could be categorized based on their localization (intracellular or extracellular) (Table 1). DNase I and DNase I-like 3 degrade excreted DNA, while DNase IIα (DNase II) and DNase III degrade mislocalized intracellular DNA. DNase I is secreted into the extracellular matrix and is the predominant endonuclease in the serum. It cleaves non-specific DNA sequences originating from apoptotic bodies, necrotic cells, and naked nucleosomes [173–175]. DNase I-like 3 (DNase IL3)—unlike its homologous DNase I—digests extracellular membrane-coated DNA, such as microparticles released by dying cells [176, 177]. DNase IIα (DNase II) is an endonuclease localized to the lysosomes of phagocytic cells. It digests membrane-engulfed DNA, generating blunt-ended DNA breaks bearing 3’ phosphates and 5’ hydroxyls [178], usually detected in the debris of apoptotic cells internalized by phagocytes [179, 180]. DNase III is a ubiquitously expressed 3’ exonuclease (*i.e.,* TREX1) localized to the cytoplasm. DNase III digests ssDNA and nicked dsDNA, including reverse transcribed DNA and RNA–DNA hybrids derived from endogenous retroelements, which may leak from lysosomes, mitochondrial and nuclear compartments [177, 181].

**Table 1:** Major DNases involved in the clearance of mislocalized DNA

DNase	Localization	Target	References
DNase I	Secreted into the extracellular matrix/serum	It digests dsDNA and ssDNA in a non-specific manner	[173]
DNase IL3	Secreted into the extracellular matrix/serum	It digests membrane-coated DNA, such as microparticles released from necrotic cells	[176, 177]
DNase II	Lysosomes of phagocytic cells	It digests membrane-engulfed DNA in an acidic condition	[178]
DNase III	Cytoplasm	It digests ssDNA, nicked dsDNA, including reverse-transcribed DNA and DNA-RNA hybrids	[181]

Although DNases are highly effective at digesting DNA, altered cell homeostasis and increased OxStr may compromise DNase efficacy. Oxidized DNA, for instance, resists TREX1-mediated degradation [182]. Of note, mtDNA, compared to nDNA, is more vulnerable to oxidative damage due to its proximity to the ETS, the primary source of ROS under physiological conditions [34]. Oka et al*.* (2012) demonstrated an important role of DNase II in digesting mtDNA [94]. In transgenic mice with a cardiac-specific DNase II deletion, accumulated mtDNA in the autolysosomes escaped degradation and activated TLR9, causing elevated levels of IL-1β and IL-6, myocarditis, and cardiac fibrosis. Therefore, the inefficacy of DNases leads to the accumulation of cytosolic or extracellular mtDNA that could engage DSRs, especially in immune sentinel cells, such as macrophages, dendritic cells (DCs), and neutrophils.

## DNA-sensing receptors and mtDNA signaling

**Fig. 5:**
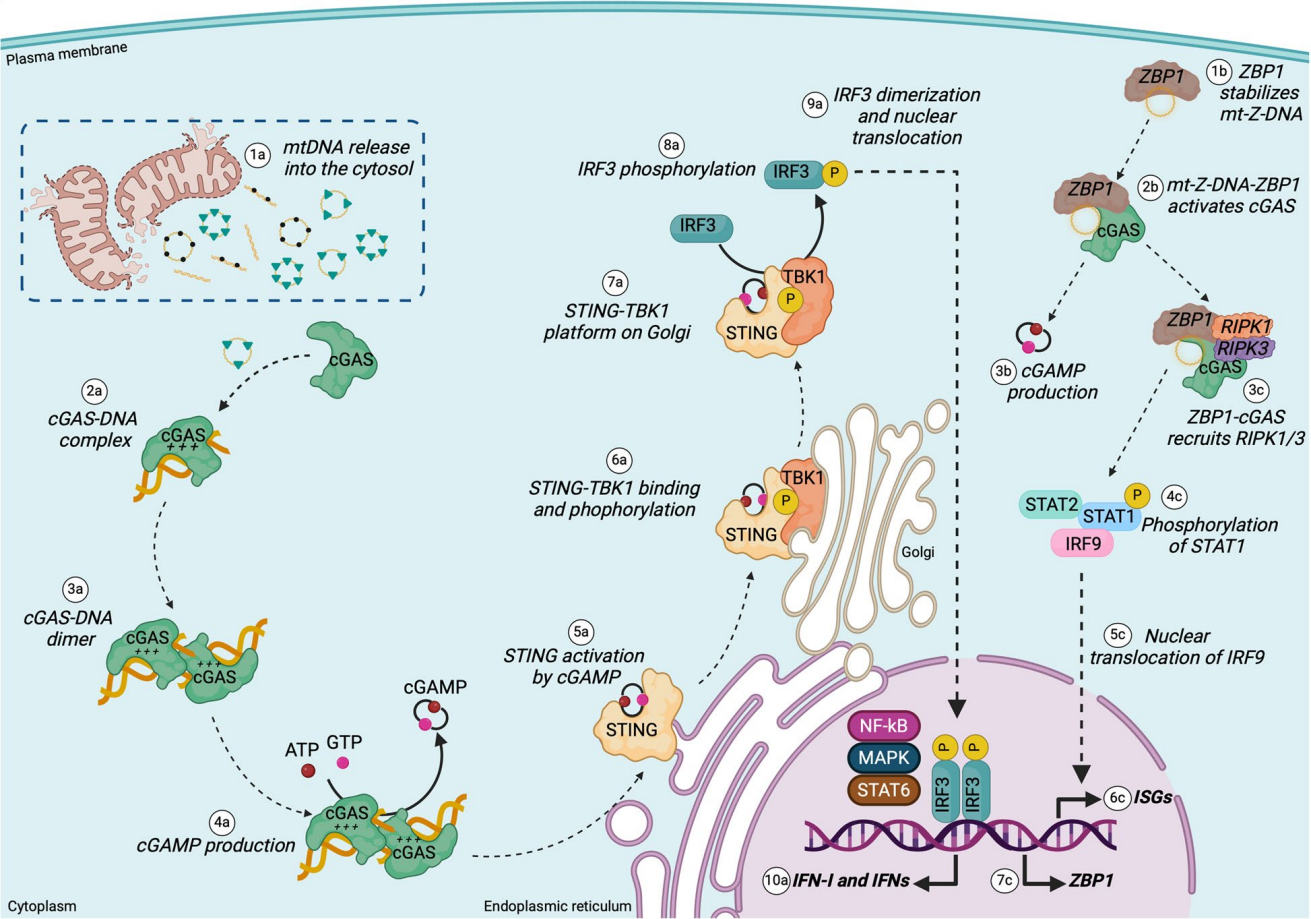
Mitochondrial DNA activates cGAS-cGAMP-STING and ZBP-cGAS pathways. Mitochondrial DNA (fragments, whole nucleoids, and oxidized mtDNA) released into the **(1a)** cytosol binds cGAS, forming a **(2a)** cGAS-DNA complex that **(3a)** after dimerization converts **(4a)** ATP and GTP in 2’,3’ cyclic GAMP (cGAMP) dinucleotide. cGAMP is a second messenger that activates **(5a)** the stimulator of interferon gene (STING), an endoplasmic reticulum (ER)-associated receptor with a binding domain that faces the cytosol. **(6a)** cGAMP-STING binds to tank-binding kinase 1 (TBK1), and TBK1 phosphorylates STING. **(7a)** On the STING-TBK1 platform in the Golgi, the C-terminal tail of STING binds the interferon regulatory factor 3 (IRF3) and **(8a)** phosphorylates it. **(9a)** Phosphorylated IRF3 dimerizes and translocates to the nucleus, where **(10a)** it promotes the expression of the type I interferon (IFN-I) and interferon stimulating genes (ISGs). Other transcription factors activated by STING are NF-kB, MAPK, and STAT6. **(1b)** ZBP1 stabilizes mt-Z-DNA released into the cytosol that facilitates the interaction of ZBP1 with cGAS. **(2b)** The DNA-protein complex activates cGAS, which catalyzes the **(3b)** production of cGAMP and also recruits **(3c)** the receptor-interacting serine/threonine-protein kinase 1 (RIPK1) and RIPK3 forming a multiprotein complex. **(4c)** These two kinases phosphorylate the signal transducer and activator of transcription 1 (STAT1) that activates **(5c)** the nuclear translocation of the interferon regulatory factor 9 (IRF9). **(6c)** IRF9 promotes the expression of the interferon-stimulating genes (ISGs), potentiating the IFN-I response. **(7c)** IFN-I *per se* positively regulates the transcription of ZBP1 by the signal transduction through type I interferon receptor

**Fig. 6:**
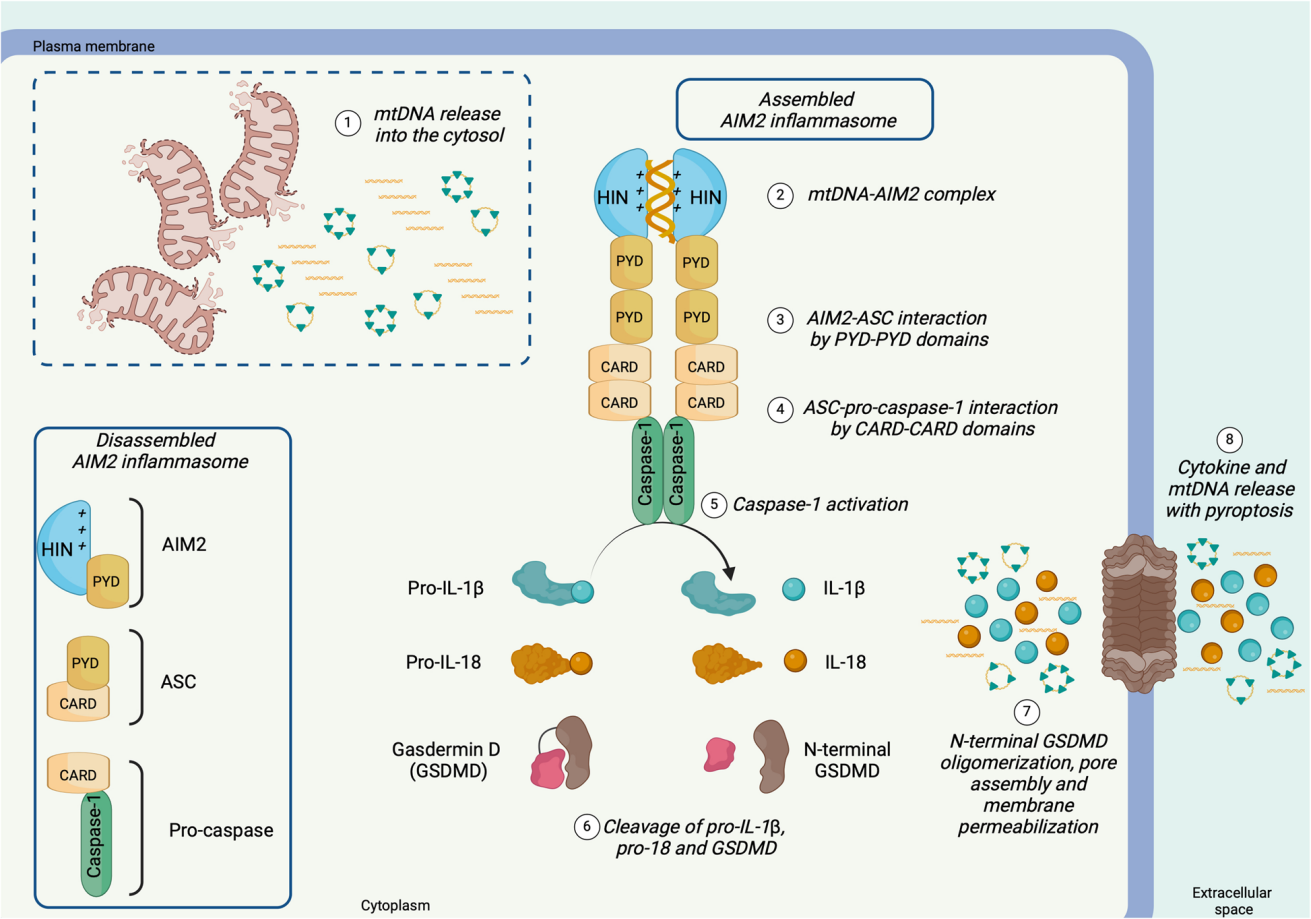
Mitochondrial DNA triggers the assembly of the inflammasome by AIM2. The absent in melanoma 2 (AIM2)-inflammasome is disassembled in physiological conditions. AIM2 protein contains two domains: the hematopoietic interferon-inducible nuclear domain (HIN) and the pyrin domain (PYD). **(1)** Mitochondrial DNA that leaks into the cytosol binds the HIN domain of AIM2, allowing its **(2)** dimerization. **(3)** AIM2 dimer interacts with the apoptosis-associated speck-like protein (ASC) by PYD domains. **(4)** ASC recruits and triggers pro-caspase-1 through the caspase activation and recruitment domains (CARD). **(5)** Active caspase-1 cleaves **(6) **pro-IL-1ß and pro-IL-18 in IL-1ß and IL-18, respectively, and generates the N-terminal domain of gasdermin D (GSDMD). **(7)** The latter oligomerizes and formes pores on the plasma membrane, by which **(8)** cytokines and other cytoplasmatic proteins are released in the extracellular environments. Protracted activation of AIM2-inflammasome leads to cell lysis by pyroptosis, which favours mtDNA release into the extracellular matrix

**Fig. 7:**
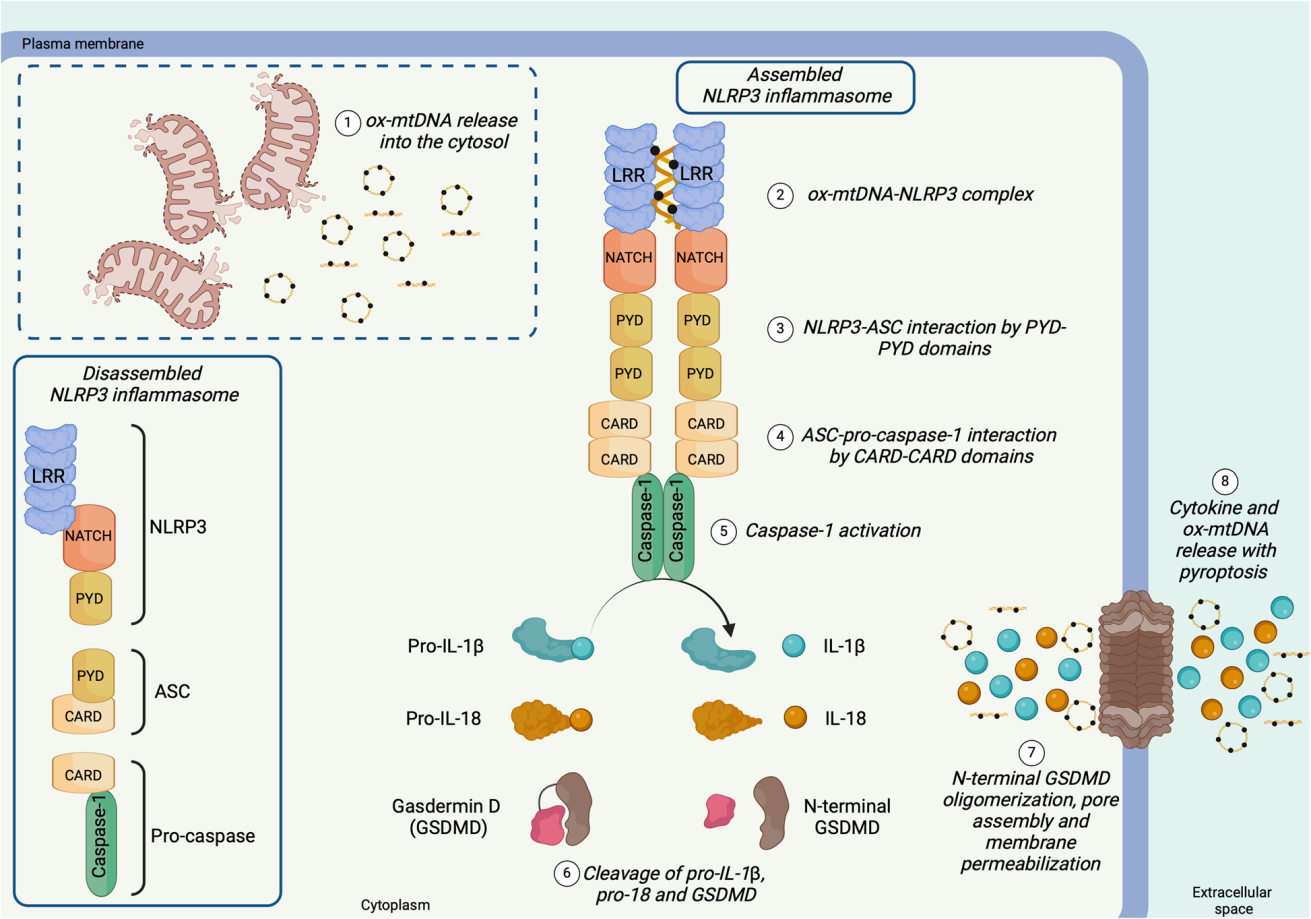
Mitochondrial DNA promotes the assembly of the inflammasome by NLRP3. The NOD-, LRR-, and PYD- domain-containing protein 3 receptor (NLRP3)-inflammasome is disassembled in physiological conditions. NLRP3 protein presents the nucleotide-binding domain leucine-rich repeat (LRR), the central nucleotide-binding domain (NACHT), and the pyrin domain (PYD). **(1)** Oxidized mtDNA (ox-mtDNA) leaking into the cytosol binds the LRR domain of NLRP3, allowing the **(2)** dimerization of NLRP3. **(3)** NLRP3 dimer interacts with the ASC apoptosis-associated speck-like protein (ASC) through PYD domains. **(4)** ASC recruits and activates pro-caspase-1 through their caspase activation and recruitment domains (CARD). **(5)** Active caspase-1 **(6)** cleaves pro-IL-1ß and pro-IL-18 in IL-1ß and IL-18, respectively, and generates the N-terminal domain of gasdermin D (GSDMD). **(7)** The N-terminal domains of GSDMD oligomerize on the plasma membrane, forming pores that allow **(8)** the release of cytokine and ox-mtDNA into the extracellular space. Protracted activation of NLRP3-inflammasome leads to cell lysis by pyroptosis, with consequent release of ox-mtDNA into the extracellular matrix

**Fig. 8:**
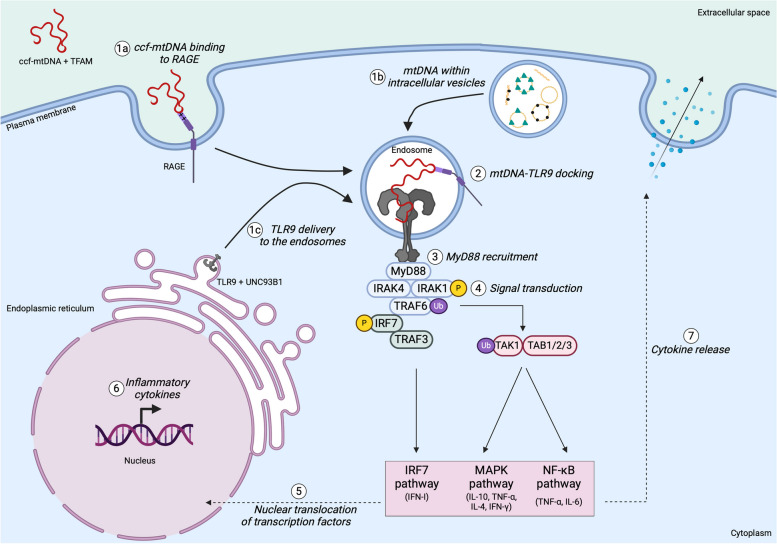
Circulating cell-free DNA and mtDNA within the intracellular vesicles trigger the immune response by TLR9. Circulating cell-free mtDNA (ccf-mtDNA) after binding to **(1a)** RAGE on the plasma membrane is endocytosed and collected into the endosomes. **(1b)** Similarly, intracellular membranes (mitochondrial-derived vesicles, autophagosomes, products of mitochondrial dynamics) containing mtDNA that escape canonical routes, fuse with endosomes. **(1c)** TLR9 from the endoplasmic reticulum is packed into COPII vesicles under the control of UNC93B1 and delivered to the endosomes, where **(2)** it homodimerizes by binding CpG motifs of mtDNA. **(3)** The TLR9 homodimer recruits myeloid differentiation primary response 88 (Myd88) that successively interacts with the interleukin-1 receptor-associated kinases 4 (IRAK4), triggering signal transduction. **(4)** IRAK4 phosphorylates IRAK1, driving a signaling cascade that, through the tumor necrosis factor receptor-associated 6 (TRAF6), activates the interferon regulatory factor 7 (IRF7) and TGF-β-activated kinase-1 (TAK1)-TGF-β-activated kinase 1-binding protein 1/2/3 (TAB1/2/3). The downstream signaling cascades lead to the **(5)** translocation in the nucleus of the transcription factors IRF7, mitogen-activated protein kinases (MAPK), and nuclear factor kappa-light-chain-enhancer of activated B cells (NF-kB). **(6)** They promote the transcription of pro-inflammatory cytokines (IL-4, IL-6, IL-10, TNF⍺, and interferons), **(7)** which are further released

The mammalian immune system has evolved several mechanisms to recognize pathogen-associated molecular patterns (PAMPs) and trigger an inflammatory response to impede pathogen propagation [183, 184]. Well-known examples are DSRs, which detect viral and bacterial DNA. However, DSRs cannot discriminate between nucleic acids from different sources and are also activated by mislocalized endogenous DNA that functions as a DAMP. The binding of DNA, including mtDNA, to DSRs depends on several factors: i) DNA conformation (ssDNA, dsDNA, DNA-RNA hybrids, B- and Z-DNA); ii) the quality of the DNA sequence (specific or non-specific, oxidized or methylated); iii) the length of the DNA sequence; and iv) the localization of the receptor (nucleus, mitochondrion, cytosol, PM). Mitochondrial DNA has been described to interact with several DSRs (Table 2): i) cGAS recognizes naked mtDNA, mtDNA packaged with TFAM, oxidized (ox-mtDNA), and potentially mtDNA-RNA hybrids [15, 35, 185]. Furthermore, cGAS can also interact with mt-Z-DNA-ZBP1 complex [40] (Fig. 5); ii) Absent in melanoma 2 (AIM2) binds naked mtDNA [186] (Fig. 6); iii) NOD-, LRR-, and PYD- domain-containing protein 3 receptor (NLRP3) docks non-oxidized and ox-mtDNA [35, 93, 187–189] (Fig. 7); iv) TLR9 recognizes unmethylated CpG regions of mtDNA [94] (Fig. 8). In the next sections, we described the signaling cascades of the above-mentioned pathways.

**Table 2:** Description of the major DNA-sensing receptors engaged by mislocalized mitochondrial DNA

DSRs	Localization	Binding	Effector	References
AIM2	• Cytosol • Mitochondria	• dsDNA • Sequence-independent	Caspase-1, IL-1β, IL-18, gasdermin D, pyroptosis	[186]
cGAS	• Cytosol • Nucleus • Plasma membrane	• dsDNA • DNA-RNA hybrid • DNA concentration-dependent and sequence-independent	STING-TBK1-IRF3	[14, 15, 83]
NLRP3	• Cytosol	• oxidized DNA • dsDNA • DNA-RNA hybrid • Sequence-independent	Caspase-1, IL-1β, IL-18, gasdermin D, pyroptosis	[93, 188, 190]
RAGE/TLR9	• Endosome	• Unmethylated CpG sequences of mtDNA	IRF7 MAPK NF-kB	[191, 192]
ZBP1	• Cytosol	• mt-Z-DNA • oxidized mtDNA • Sequence-independent	cGAS-STING and RIPK1/3- STAT1	[40, 193]

### cGAS-cGAMP-STING and ZBP1-cGAS

The cGAS-STING pathway is highly conserved across vertebrates and is essential for the immune response [194]. Transgenic mice lacking cGAS are highly susceptible to bacterial and viral infections [183]. cGAS-STING signaling upregulates the nuclear factor kappa-light-chain-enhancer of activated B cells (NF-κB), signal transducer activator of transcription 6 (STAT6), the mitogen-activated protein kinases (MAPKs) and cytokine cascades [195, 196]. It also controls the proliferation of T lymphocytes [197], initiates cell death in B lymphocytes [198, 199] and monocytes [197, 200], and promotes autophagy-dependent ferroptosis [130] and senescence [201–203].

cGAS is an intracellular enzyme that is localized to the cytosol, nucleus, and PM [204–206]. It binds the sugar-phosphate DNA backbone in a sequence-independent but concentration-dependent fashion by recognizing its positively charged residues [207, 208] (Fig. 5). Upon binding to dsDNA, cGAS assembles into a 2:2 cGAS-dsDNA complex. This dimer converts ATP and guanosine 5’-triphosphate (GTP) to 2’, 3’ cyclic GAMP (cGAMP), an unusual cyclic dinucleotide [209, 210]. 2’, 3’cGAMP serves as a second messenger by binding and activating STING protein, an endoplasmic reticulum-associated receptor with a binding domain that faces the cytosol [194]. The cGAMP-STING complex translocates from the ER to the Golgi intermediate compartment (ERGIC) via the cytoplasmic coat protein complex II (COPII) [211, 212]. In the ERGIC, STING binds to tank-binding kinase 1 (TBK1) and undergoes palmitoylation. TBK1 directly phosphorylates STING, and the C-terminal tail of STING binds to the transcription factor interferon regulatory factor 3 (IRF3). Acting as a platform, STING mediates the phosphorylation of IRF3 by TBK1. Phosphorylated IRF3 dimerizes and translocates to the nucleus, promoting the transcription of IFN-I and several ISGs [213]. IFN-I gene family comprises 13 subtypes of IFN-α and a single IFN-β, that mount a broad antiviral defense [214].

The cGAS-cGAMP-STING pathway is activated by a variety of stimuli detecting any dsDNA that accumulates in the cytoplasm. cGAS docks both short dsDNAs (20 bp) and long dsDNAs (~ 45 bp), although longer dsDNAs induce its stronger enzymatic activity by forming more stable dimers [207, 208, 215]. Viral, retroviral, and bacterial DNA [183, 216], cytoplasmic mtDNA and ox-mtDNA [15, 35], extracellular DNA released from EVs [217], or nDNA released from the nucleus due to chromosome instability [218–220] can bind cGAS. Additionally, histone U93 (HU) and TFAM (bacterial and mitochondrial nucleoid proteins, respectively), or the high mobility group box 1 protein (HMGB1), strongly stimulate cGAS-DNA binding by facilitating conformational changes in the DNA that favour cGAS dimerization [221]. Of note, STING is also activated by cGAS-independent stimuli, such as ER stress [222, 223], viral liposomes [224], and 3’, 5’cyclic dinucleotides (cyclic diAMP, cyclic diGMP, and cyclic cGAMP). The latter originate from bacteria and bind directly to STING with tenfold lower affinity than 2’, 3’cGAMP [194, 225, 226]. Interestingly, 2’, 3’cGAMP crosses gap junctions, acting as a paracrine signal by activating STING and antiviral responses in neighboring epithelial cells that did not accumulate cytosolic DNA upfront [227]. cGAS signaling is tightly regulated. Without cell stress and infection and after initiation, the cGAS steady state depends on its autophagic degradation [228, 229]. Upon activation, its activity is governed by post-translational modifications [184, 230].

Two independent studies showed for the first time that mtDNA could engage cGAS (Fig. 5). Mitochondrial permeabilization caused by BAX and BAK, combined with caspase inhibition, resulted in the release of mtDNA that docked cGAS [14, 83]. On the contrary, activation of the apoptotic caspases blocked cGAS/STING signaling by directly cleaving key proteins, including cGAS and IRF3 [231]. These findings indicated that apoptosis is required to prevent an immune response that would otherwise be activated when mtDNA binds to cGAS in the cytosol. West and colleagues confirmed the molecular link between mtDNA release, cGAS, and IFN-I expression [15]. They showed that aberrant mtDNA packaging caused by genetic and pharmacological downregulation of TFAM promoted mtDNA release into the cytosol, where it induced IFN-I expression by the cGAS-STING signaling.

Recently, West’s group identified a cooperative mechanism of mtDNA sensing between cGAS and ZBP1, a double-stranded Z-RNA and Z-DNA receptor that activates several pathways [232, 233]. Authors observed that ZBP1 ablation abrogated the expression of the ISGs in TFAM Het MEFs, human cells, and in a mouse model of cardiotoxicity induced by doxorubicin [40]. They demonstrated that i) mitochondrial genome instability promotes mt-B-DNA to mt-Z-DNA transition and release; ii) cytosolic mt-Z-DNA is stabilized by ZBP1, whereas mt-B-DNA presumably remains still immunostimulatory by activating cGAS; The mt-Z-DNA-ZBP1 duo by the RIP homotypic interaction motif (RHIM) domain of ZBP1 interacts with cGAS forming a complex; iii) mt-Z-DNA-ZBP1-cGAS complex promotes cGAMP synthesis. It also engages RIPK1 and RIPK3 in the absence of MLKL activation, augmenting the phosphorylation of the signal transducer and activator of transcription 1 (STAT1), potentiating the IFN-I response to the mtDNA instability (Fig. 5). Of note, the crosstalk between cGAS and ZBP1 is further enhanced by the fact that IFN-I, a product of the cGAS activation, binds to the type I interferon receptor (IFNAR) and upregulates ZBP1 expression. Importantly, the study did not define the specific Z-DNA sequences of mtDNA that are stabilized by ZBP1, and it did not exclude the concomitant presence of cytosolic nuclear Z-DNA that may be derived by the genome instability caused by mtDNA stress [11, 234], calling for further investigations. Overall, these results indicate that cytosolic mtDNA is a mitochondrial stress messenger that mounts an innate immune response by cGAS-STING and ZBP1-cGAS complex, independently of the presence of viral or bacterial DNA.

### AIM2 inflammasome

Another protein involved in the sensing of mislocalized mtDNA is absent in melanoma 2 (AIM2), which activates the inflammasome signaling. AIM2 is predominantly localized to the cytosol, where it recognizes dsDNA, but data also suggested that it localizes to the mitochondria [235]. It belongs to the absent in melanoma like-receptor (ALR) family, which also includes the interferon-inducible 16 (IFI16), myeloid nuclear differentiation antigen (MNDA), and interferon-inducible X (IFIX) [236]. AIM2, like all the ALR family members, contains an evolutionary conserved pyrin domain (PYD) involved in protein–protein interactions, together with a hematopoietic interferon-inducible nuclear domain (HIN) that binds to DNA. The C-terminal domain (HIN-200) of AIM2 has two tandem β barrels with positively charged amino acids that form an oligonucleotide/oligosaccharide-binding fold that interacts with the sugar-phosphate DNA backbone in a sequence-independent manner [237] (Fig. 6). The dsDNA (~ 300 bp) binding induces AIM2 dimerization, triggering the formation of the inflammasome, a multimeric protein platform that leads to caspase-1 activation, cytokine release, and cell lysis [237, 238]. AIM2 is liberated from its autoinhibited state by binding to DNA. The binding induces conformational changes, which allow the interaction between AIM2 and the apoptosis-associated speck-like (ASC) protein through their PYD domains. Activated ASC recruits pro-caspase-1 via the interaction of their respective caspase activation and recruitment domains (CARDs). The activated caspase cleaves pro-IL-1β and pro-IL-18 into their mature forms (IL-1β and IL-18, respectively) [239–241] and cleaves GSDMD, promoting the formation of pores on the PM by its N-terminal. The resulting pores enable cytokine and cytoplasmic efflux into the extracellular environment, causing macrophage infiltration and phagocytosis of cell debris [242].

Two seeding studies speculated on the role of mtDNA in the AIM2 activation. A report showed that BMDMs, previously primed with LPS, increased the secretion of IL-1β and IL-18 when stimulated with ccf-DNA (containing high levels of mtDNA) isolated from patients with type 2 diabetes (T2D) or with synthetic dsDNA (polydA:dT) [186]. The authors concluded that elevated levels of ccf-mtDNA observed in T2D patients contributed to the chronic inflammation via AIM2 inflammasome since they excluded the involvement of NLRP1 and -3. However, the mechanism of ccf-mtDNA efflux to the cytosol to engage AIM2 was not established. Another study described the release of mtDNA into the cytosol and AIM2 inflammasome activation in cardiomyocytes in an aggravated post-infarct mouse model of T2D [243], although direct evidence of AIM2-mtDNA docking was not provided, indicating that further studies are still needed.

Interferon inducible 16 (IFI16)—another member of the ALR family – has also been shown to interact with DNA and activate the inflammasome cascade [236]. IFI16 has three domains: a PYD and two linked HIN domains (HINA and HINB) [244, 245]. It localizes to both the nucleus and cytoplasm and detects dsDNA and ssDNA [246, 247]. Like AIM2, the dsDNA-IFI16 complex activates the inflammasome. It has been shown that IFI16 binds mislocalized mtDNA in vitro [248], however, compared to the other DSRs, the activation of IFI16 by mtDNA remains investigated only in a few pathological conditions [249].

### NLRP3 inflammasome

NLRP3 is a cytosolic DSR that triggers the inflammasome by binding non-oxidized and ox-mtDNA [35, 188, 189]. However, whether mtDNA is released upstream of NLRP3 (acting as an NLRP3 inflammasome activation signal) or downstream (because of its activation) is still unclear [250, 251]. NLRP3 is a member of the nucleotide-binding domain leucine-rich repeat (LRR)-containing receptor (NLR) family. It is composed of three domains: a PYD for protein–protein interactions, a central nucleotide-binding oligomerization domain (NACHT) for self-oligomerization, and a LRR domain for stimulus recognition [252]. The NLPR3 mechanism to activate the inflammatory response closely mimics that of AIM2. NLRP3 protein binds to ASC through their respective PYDs. ASC then binds pro-caspase-1 through their CARD domains. NLRP3, ASC, and pro-caspase-1 assemble to form a multiprotein complex known as the NLRP3 inflammasome (Fig. 7). Activated caspase-1 cleaves pro-IL-1β and pro-IL-18 into their mature pro-inflammatory cytokines, which mediate a specific immune response, leading to cell death by pyroptosis [250, 252].

It has been demonstrated that ox-mtDNA released into the cytosol activates the NLRP3 inflammasome [35, 188]. Additionally, ATP and nigericin, which promote mtDNA oxidation and release into the cytosol, have been shown to trigger NLRP3 activation independently of AIM2 in BMDMs. An interesting study reported that LPS treatment promoted mtDNA replication in macrophages by the upregulation of cytidine/uridine monophosphate kinase 2 (CMPK2), a rate-limiting enzyme involved in the synthesis of deoxyribonucleotides [190]. Under conditions of OxStr, the newly synthesized mtDNA fragments were oxidized (8-OH-dG) and released into the cytosol, with the activation of NLRP3 inflammasome. Other studies have shown that the accumulation of damaged mitochondria caused by increased ROS impaired mitophagy flux in macrophages and induced release of ox-mtDNA into the cytosol, leading to the activation of NLRP3 inflammasome [93, 187]. Mechanistically, Parkin cleavage by caspase-1 inhibits mitophagy, amplifying mitochondrial stress and mtDNA release into the cytosol [253, 254]. These observations indicate that cytosolic ox-mtDNA is not only involved in NLRP3 inflammasome activation, but it accumulates because of caspase-1 activation, creating a vicious cycle that perpetuates inflammation.

### Priming and activation of the AIM2- and NLRP3-dependent inflammasome

Triggering the formation of the AIM2- and/or NLRP3-dependent inflammasome involves two main signals: priming and activation. Priming aims to increase the expression of AIM2, NLRP3, pro-IL-1β, and pro-IL-18, and can be transcription-dependent or -independent [252]. Transcription-dependent priming is achieved by toll-like, TNF, and IL-1 receptors that recognize PAMPs and DAMPs to activate the nuclear factor kappa-light-chain-enhancer of activated B cells (NF-κB) [255]. NF-κB promotes AIM2, NLRP3, pro-IL-1β, and pro-IL-18 expression through interactions with myeloid differentiation primary response 88 (MyD88) and interleukin-1 receptor-associated kinases 1 (IRAK1) and 4 (IRAK4). Transcription-independent priming is achieved through deubiquitination of AIM2 and NLRP3, which increases their stability and dimerization. This process is controlled by several proteins, including BRCA1/BRCA2-containing complex subunit 3 (BRCC3), a TIR-domain-containing adapter-inducing interferon-β (TRIF), IRAK1-activated deubiquitinases, and ubiquitin specific peptidase 21 (USP21) [251, 256].

Activation is governed by molecules and/or cellular events such as DAMPs, PAMPs, extracellular ATP, Ca^2+^ influx, K^+^ efflux, phagosome instability, nigericin (a microbial toxin), mtROS and cardiolipin translocation [250–252, 255]. Unnecessary and harmful activation is prevented through priming inhibition by several mechanisms, including microRNA and nitric oxide signaling [255]. Based on their roles, cytosolic mtDNA and ccf-mtDNA could be considered priming and activator molecules.

### TLR9

The toll-like receptors (TLRs) represent another class of ten human well-characterized proteins involved in the initiation of pro-inflammatory signaling cascades in response to various antigens, including foreign and endogenous mislocalized mtDNA [105, 120]. TLR9 detects unmethylated CpG motifs existing in bacterial DNA and mtDNA [257, 258] (Fig. 8). TLR9 is a transmembrane receptor whose signaling is initiated through a Toll/IL-1R resistance (TIRs) domain. In the absence of DNA antigens, TLR9 is localized to the ER, although it has also been shown to localize on the cell surface of several cell types, including RBCs [259]. Once DNA antigens are detected and endocytosed (Sect. "RAGE-mediated endocytosis tethers the ccf-mtDNA-TLR9 interaction"), the trafficking protein Unc-93 homolog B1 (UNC93B1) transports TLR9 to the DNA-containing endosome, and the N-terminal ectodomain of TLR9 gets cleaved by asparagine endopeptidase and cathepsins [260–262]. Of note, TLR9 can also bind mtDNA enclosed within the intracellular membranes (mitochondrial-derived vesicles, autophagosomes) that escape canonical routes and fuse with the endosomes [191]. TLR9 homodimerizes when the CpG motifs in the DNA act like a bridge binding to the LRR of the cleaved N-terminal fragment and to the LRR of the C-terminal fragment of an adjacent TLR9 [263]. The formation of a ligand-dependent homodimer promotes the recruitment of MyD88 to mediate protein–protein interactions between TLR9 and subsequent signal transduction components. The first of these interactions involves IRAK4. IRAK4 forms an oligomeric complex with TLR9 and MyD88 called the Myddosome. Once IRAK4 is activated through *trans*-autophosphorylation*,* it phosphorylates IRAK1 [258, 264]. Successively, IRAK4 and IRAK1 interact with the tumor necrosis factor receptor-associated 6 (TRAF6), a ubiquitin ligase that catalyzes lysine 63-linked polyubiquitination of itself and the TGF-β-activated kinase-1 (TAK1) complex. TAK1 then associates with TGF-β-activated kinase 1-binding protein 1 (TAB1), TAB2, and TAB3 to activate the NF-κB and MAPK pathways promoting an inflammatory response. Specifically, the NF-κB pathway activates the transcription of TNF-α and IL-6, while the MAPK pathway activates TNF-α, IL-4, IL-10, and interferon-γ (IFN-γ) expression [258, 264]. TLR9 also induces IFN-I expression by activating the interferon regulatory factor 7 (IRF7) pathway through IRAK1 and TRAF3 [258, 265]. Ccf-mtDNA-TLR9 signaling transduction through the NF-κB, MAPK, and IRF7 triggers an inflammatory response [120, 191, 266].

### RAGE-mediated endocytosis tethers the ccf-mtDNA-TLR9 interaction

The activation of TLR9 by ccf-mtDNA is well-documented. Julian and colleagues (2012) were the first to measure the levels of IFN-α released by plasmacytoid DCs exposed to a purified mitochondrial fraction from necrotic HepG2 cells, with and without treatment with DNase [192]. They showed that increased IFN-α levels were primarily dependent on the presence of mtDNA. Using a competitive TLR9 inhibitor, they demonstrated that TLR9 was mediating the IFN-α upregulation. The mechanism by which ccf-mtDNA enters the cell and is presented to TLR9 is unclear. Some evidence suggests that the majority of ccf-mtDNA in the bloodstream may be encapsulated in extracellular vesicles (EVs), including exosomes [279, 280] or whole mitochondria, that would not make mtDNA directly accessible to receptors [112, 281]. However, Sirois et al. (2013) demonstrated that extracellular DNA binds the receptor for advanced glycation end-product (RAGE) at the PM [191] (Fig. 8). By solving the crystal structure of the RAGE-DNA complex, they found that RAGE forms a positively charged binding pocket that interacts with the negatively charged sugar-phosphate backbone of DNA. Furthermore, they demonstrated that RAGE binds to DNA in a sequence-independent manner, followed by the DNA-RAGE complex translocation to both early and late endosomal compartments. Co-immunoprecipitation of TLR9 and RAGE from cell lysates before and after exposure to CpG DNA indicated that RAGE-associated DNA is predominantly delivered to TLR9, and that these receptors may interact simultaneously with the same ligand. While RAGE can activate NF-κB through its signaling domain, its interaction with CpG DNA enhanced NF-κB signaling in a TLR9-dependent manner. The authors also noted that TLR9 was activated in the absence of RAGE, indicating that there may be additional mechanisms of CpG DNA delivery to TLR9 [191].

### TFAM modulates mtDNA immunogenicity

The role of TFAM haploinsufficiency in triggering mtDNA instability was previously discussed (Sect. "Mitochondrial DNA instability"). In this section, we describe how TFAM modulates mtDNA immunogenicity and how it is involved in nucleoid-phagy. It has been shown that HMG box 1 (HMGB1) proteins elicit an inflammatory response independent of their DNA ligands by binding directly to TLR4 and RAGE [282]. Because TFAM is structurally and functionally homologous to HMGB1, it also directly promotes sterile inflammation by binding TLR4 and RAGE [282]. Although TFAM is not required for RAGE-dependent DNA uptake, it has been proved that CpG DNA binding to HMGB1 proteins activated TLR9 and elicited a greater immune response than the binding of HMGB1 alone [283]. Similarly, higher IFN level was observed when DCs were co-treated with purified TFAM and CpG DNA compared to TFAM treatment alone. On the contrary, decreased levels of IFN were observed in cells treated with either RAGE or TLR9 inhibitors [192, 257]. There are two reasons by which TFAM augments TLR9 activation. First, TFAM has a high affinity for heparin sulfate, which is required for RAGE-dependent signaling [257, 284]. Second, TFAM bends and stabilizes ccf-mtDNA, enhancing its interaction with TLR9 [285, 286], which prefers curved DNA backbones and U-turns as its ligand, similar to cGAS [221]. These findings indicate that TFAM-associated ccf-mtDNA enhances the activation of TLR9 through RAGE-mediated endocytosis [287].

Recently, Liu et al*.* (2024) showed that TFAM plays a direct role in the degradation of mtDNA by nucleoid-phagy to avoid/curtail the cGAS-STING inflammatory pathway [19]. They demonstrated that: i) blocking autophagy by knocking out the autophagy-related protein 7 (ATG7) or by pharmacological inhibition (using bafilomycin A1), increased the cytoplasmic accumulation of the mitochondrial nucleoids induced by H_2_O_2_ in HeLa cells, and in THP1 cells treated with ATP and LPS; ii) cytosolic mtDNA colocalized and co-immunoprecipitated with TFAM and LC3B; iii) the mature TFAM protein has two LC3 interacting region (LIR) motifs, LIR1 and LIR2, with LIR2 that binds LC3B to mediate degradation of mtDNA via the autophagic lysosomal pathway; iv) cells expressing LIR2 deficient TFAM failed to degrade the nucleoids with accumulation of cytosolic mtDNA and increased STING/IRF3/IFN-β signaling; v) the impasse of TFAM-LC3-dependent nucleoid-phagy causes mtDNA leakage into the cytosol (Fig. 3). Altogether, these findings identify a new role of TFAM as a main player involved in the sequestration of cytosolic mtDNA by autophagy, avoiding triggering the cGAS-STING inflammatory pathway.

## mtDNA signaling via DSRs in vascular and metabolic diseases

Mitochondrial content and activity are extremely high in vascularized organs like the liver and heart, indicating the pivotal metabolic and bioenergetic roles [288]. Cytosolic and ccf-mtDNA activating DSRs have been reported in samples isolated from patients with vascular and metabolic diseases and in several experimental models, including type II diabetes mellitus (T2DM), obesity, cardiac and liver diseases.

### cGAS-STING

The activation of cGAS-STING by mtDNA has been documented in endothelial cells (ECs) and mouse models of vascular diseases. Two groups observed that palmitic acid induced mtDNA leakage into the cytosol, which activated cGAS-STING-IRF3 in ECs. This signaling cascade caused vascular inflammation, reduced cell proliferation, migration, and angiogenesis [289, 290]. Cellular proliferation was inhibited by the IRF3-induced expression of mammalian step20-like kinase 1 (MST1) that deactivated the transcription factor yes-associated protein (YAP) [289]. The crosstalk between the mtDNA-cGAS-STING-IRF3 axis and YAP dysregulation was also described in other reports. In a mouse model of severe vascular injury induced by LPS (sepsis), activated GSDMD formed mitochondrial pores in ECs, allowing the release of mtDNA into the cytosol. The cytosolic mtDNA initiated cGAS-STING-IRF3 signaling, leading to the suppression of vascular regeneration [167]. IRF3-signaling inhibited the nuclear translocation of the transcription factor YAP1 and indirectly blocked cyclin D-mediated cell proliferation to foster cell senescence. Downregulation of cGAS by siRNA restored endothelial proliferation, suggesting that cytosolic mtDNA decreased EC proliferation during vascular inflammation. The mtDNA-cGAS-STING-IRF3 axis triggered by supplementation with palmitic acid also increased the expression of intercellular adhesion molecule 1 (ICAM-1), stimulating the adhesion of monocytes to ECs, a hallmark of endothelial inflammation [290].

A recent study links aberrant mtDNA synthesis in human and mouse macrophages with cGAS-STING activation and the progression of atherosclerotic plaque [291]. The authors showed that the expression of the vascular cell adhesion molecule-1 (VCAM-1) was increased in macrophages located in the atherosclerotic plaques. Mice lacking Vcam1 in macrophages exhibited smaller atherosclerotic plaques and necrotic core areas compared to WT. High VCAM-1 expression in macrophages increased mtDNA synthesis, its oxidation and fragmentation, which led to the activation of cGAS-STING inflammation, raising the plaque burden of mice on atherogenic diet [291]. Although this study identified the pro-inflammatory effect of mtDNA in atherosclerotic macrophages through the cGAS–STING pathway, authors did not directly investigate how mtDNA escaped to the cytoplasm, how it docked cGAS, and neither how mtDNA got oxidized.

There are evidences for mtDNA triggering cGAS-STING signaling in metabolic disorders. STING activation appears to be a key player in obesity-related inflammation. In a mouse model of obesity induced by a high-fat diet, *Sting* KO partially prevented endothelial inflammation and the infiltration of macrophages in the vessels of the adipose tissue. STING deficiency also ameliorated body weight, free fatty acids in the plasma, insulin resistance, and glucose intolerance [290]. In a similar model of obesity, it has been documented mtDNA release into the cytosol of adipocytes and macrophages, followed by cGAS-STING activation [267]. The authors observed that the mitochondria-localized disulfide bond-forming oxidoreductase A-like protein (DsbA-L)—a glutathione-S-transferase kappa 1 enzyme (GSTK1) and a key regulator of adiponectin biosynthesis—was downregulated in obese mice [292]. Its downregulation increased mtROS production, decreased ∆Ψ_mt_, increasing mtDNA release into the cytosol. Overexpression of DsbA-L suppressed mtDNA-cGAS-STING signaling and reduced the levels of inflammatory cytokines in the serum [267].

### AIM2/NLRP3 inflammasome

Several studies showed that cytosolic mtDNA drives an AIM2-mediated inflammatory response in metabolic disorders, including diabetes. In a mouse model of T2DM, AIM2, caspase-1 and IL-18 were found to be upregulated in the infarct regions of the hearts that underwent coronary artery ligation [243]. These mice presented an altered inflammatory response resulting in fibrosis, with increased levels of type I macrophages (M1 macrophages, pro-inflammatory) and decreased type II macrophages (M2, pro-reparative). The inflammatory phenotype was caused by impaired mitophagy that led to the accumulation of mitochondria in the autophagosomes of cardiomyocytes. The consequent release of mtDNA into the cytosol activated the AIM2 inflammasome with IL-1β and IL-18 secretion, which reprogrammed M2 macrophages to M1. The crosstalk between cardiomyocytes and macrophages was driven by cytosolic mtDNA [243]. As clinically relevant, high levels of IL-1β and ccf-mtDNA were observed in the plasma of patients with T2DM, with the latter thought to activate the AIM2 inflammasome in macrophages [186].

Elevated cholesterol level, another risk factor associated with cardiovascular diseases, increases ROS production and promotes the accumulation of dysfunctional mitochondria [293]. It has been shown that cholesterol supplementation in LPS-activated BMDMs induced the release of mtDNA into the cytosol, but not nDNA, followed by secretion of IL-β [294]. Activated macrophages utilize the enzyme cholesterol 25-hydroxylase (Ch25h) to decrease cholesterol levels and produce oxysterol 25-hydroxycholesterol (25-HC). Interestingly, *Ch25h* KO macrophages exhibited dysfunctional mitochondria with mtDNA release into the cytosol, AIM2 inflammasome activation, and IL-β secretion. This phenotype was rescued in cells overexpressing Ch25h and in the double *Ch25h/AIM2* KO cells [294], emphasizing the role of cytosolic mtDNA in the disorders caused by cholesterol.

Fatty acid accumulation and oxidation have been shown to activate the NLRP3 inflammasome in hepatic cells. In a mouse model of nonalcoholic steatohepatitis (NASH), Kupffer cells (resident macrophages) exhibited increased mtROS production, decreased ∆Ψ_mt_, increased mtDNA release into the cytosol and NLRP3 inflammasome activation [295]. Similar results were found in mice fed with a diet containing high levels of linoleic acid [296, 297], and macrophages isolated from diabetic mice [298]. NLRP3 inflammasome activation has been also shown to be driven by leakage of mtDNA into the cytosol in livers isolated from a rat model of T2D, in which hepatic insulin resistance was caused by arsenic administration [299], and in cardiomyocytes during myocardial ischemia [300].

### TLR9

Cardiac injury is exacerbated by RAGE/TLR9 signaling activated by mtDNA. Two studies reported elevated levels of ccf-DNA in the bloodstream of mice following myocardial I/R injury [118, 301]. One study detected elevated levels of ccf-mtDNA [118], whereas the other observed increased levels of total ccf-DNA (without discrimination between nDNA and mtDNA) and extracellular HMGB1 [301]. In the same studies, hearts perfused with DNase I or a monoclonal antibody against HMGB1 during I/R, resulting in mtDNA destabilization and reduced activation of RAGE/TLR9 signaling, were found to decrease infarct size [118, 301]. On the contrary, perfusion with both recombinant HMGB1 (rHMGB1) and purified mtDNA exacerbated the infarct size [301]. Interestingly, hearts treated separately with either rHMGB1 or mtDNA resembled that of the control, demonstrating the synergistic effect of stabilized mtDNA in activating TLR9. To confirm that rHMGB1 and mtDNA were increasing the infarct size by TLR9 signaling, the same experiments were repeated using *Tlr9* KO and *Rage* KO mice. This time, no differences in the infarct size between the untreated, DNase I-treated, *Tlr9* KO, and *Rage* KO hearts were observed [118]. Similarly, ccf-mtDNA was also found high in the conditional medium of cultured blood cells obtained from a cohort of patients with atrial fibrillation [302]. It also stimulated cytokine expression by TLR9 signaling in macrophages. These studies demonstrate that mtDNA released from necrotic cardiomyocytes exacerbates cardiac injury through RAGE/TLR9 signaling.

## mtDNA signaling via DSRs in kidney diseases

Cytosolic and extracellular mtDNA play a role in the acute and chronic injuries of the kidney by binding DSRs and amplifying primary damages.

### cGAS-STING

The immune response via the cGAS-STING pathway is involved in the progression and severity of kidney injuries. Two independent groups demonstrated that mtDNA activates cGAS-STING in acute and chronic kidney injuries (AKI and CKI, respectively). Maekawa et al*.* (2019) showed that patients affected by AKI presented tubular mitochondrial dysfunction and inflammation [303]. In a mouse model of AKI induced by cisplatin, they observed mitochondrial damage leading to mtDNA leakage into the cytosol via BAX/BAK pores. The consequent production of cytokines caused by the activation of the mtDNA-cGAS-STING signaling promoted neutrophil infiltration and tubular inflammation. Genetic and pharmacological ablation of STING blunted inflammation and provided partial protection against AKI, suggesting that other mechanisms may trigger inflammatory pathways, including TLR9 activation by mtDNA [304]. Similar to Maekawa’s study, Chung and colleagues (2019) found decreased expression of TFAM and mtDNA-encoded genes with increased IL-1β and IL-6 expression in kidneys from patients with CKI and mouse models of kidney fibrosis [37]. The authors observed a similar phenotype in tubule-specific *Tfam* KO mice. In those mice, aberrant packaging of mtDNA fostered its efflux into the cytosol, where it engaged cGAS-STING, promoting cytokine expression, immune cell recruitment, and kidney fibrosis. Again, selective ablation of STING attenuated the kidney fibrotic phenotype [37]. Recently, impaired mitophagy, cytosolic release of mtDNA, and cGAS-driving inflammation were shown in a mouse model recapitulating human ADTKD-UMOD [102].

### AIM2/NLRP3 inflammasome

The AIM2/NLRP3 inflammasomes are also a key component in the development and progression of chronic kidney disease (CKD). Immunofluorescence of kidney tissues isolated from patients with CKD displayed high levels of AIM2 and inflammation markers, whereas AIM2 deficiency attenuated renal inflammation and fibrosis in a mouse model of unilateral ureteral obstruction [305]. By using intravital microscopy and cultured cells, authors demonstrated that macrophages engulfing necrotic cells activated AIM2 inflammasome with IL-1β secretion. Treatment with DNase I attenuated IL-1β levels, suggesting that extracellular DNA was the predominant signal contributing to the phenotype. However, researchers did not analyze the single effect of mtDNA or nDNA, nor their synergic role in promoting AIM2 inflammasome activation, warranting further investigations [305]. Two mouse models—one mimicking proteinuria in renal tubular injury, the second caused by nephrectomy – also suggested a prominent role for mitochondrial dysfunction and NLRP3 inflammasome activation in the pathogenesis of CKD [306, 307].

### TLR9

The TLR9-activated inflammation also seems critical in AKI. Mitochondrial OxStr, swelling, and loss of cristae were found in the proximal tubules of mice with septic AKI induced by cecal ligation and puncture [304]. Mitochondrial dysfunction was accompanied by high levels of ccf-mtDNA and cytokines in the mouse plasma and peritoneal cavities. To understand the role of mtDNA in AKI pathogenesis, WT and *Tlr9* KO mice were intravenously injected with exogenous mitochondrial debris (EMD). The immune profile and cellular damage of WT mice treated with EMD were similar to that of mice with septic AKI, whereas cytokines were reduced in *Tlr9* KO mice and in mice that received EMD previously digested with DNase [304]. These results suggest a direct role of ccf-mtDNA in septic AKI pathogenesis by TLR9 signaling.

## mtDNA signaling via DSRs in lung diseases

Several reports highlighted the involvement of the cytosolic and ccf-mtDNA in the activation of the immune response in acute and chronic lung diseases.

### cGAS-STING

High levels of ccf-DNA in the plasma and elevated levels of C-X-C Motif Chemokine Ligand 10 (CXCL10) were found in the sputum of patients with silicosis [277]. Increased STING expression was also reported in the lung sections derived from patients with fibrotic interstitial lung disease. Using a mouse model of silicosis, authors showed that intratracheal administration of silica increased mtROS production in lung DCs, triggering the release of mtDNA into the cytosol that by cGAS-STING signaling induced IFN-I expression. They also demonstrated that silica induced STING-dependent apoptosis in DCs and necroptosis in macrophages, with both contributing to the extracellular release of dsDNA. Treatment with DNase I inhibited silica-induced STING activation and IFN-I response [277].

Increased ccf-mtDNA level was observed in the bronchoalveolar lavage (BAL) and plasma of patients with IPF and was positively correlated with disease progression and fatal outcomes [308]. Schuliga et al. (2020) demonstrated that mtDNA is released into the cytosol and conditional medium of fibroblasts isolated from the lungs of IPF patients, together with the upregulation of cGAS [309]. Furthermore, when mtDNA was added to the cell growth medium of healthy fibroblasts, expression of senescent markers—a hallmark of IPF—were increased. Treatment with DNase I, pharmacological and genetic inhibition of cGAS decreased the expression of senescence markers. The same group also observed that expression of the cyclin-dependent kinase inhibitor 1A (CKD1A; a marker of senescence), cGAS, and phosphorylated STING level were increased in the epithelial cells of the lungs isolated from patients with IPF [268]. Cell population analysis revealed that senescent alveolar epithelial cells (type I and II) released mtDNA into the cytosol and extracellular space. Treatment with rotenone further triggered mtDNA release and increased the expression of senescence markers, including IL-6, IFN-β, and TGF-β, whereas pharmacological inhibition of cGAS diminished them. These findings demonstrate that mislocalized mtDNA contributes to the onset of a senescent phenotype in IPF.

DNA release into the cytosol and cGAS activation seem to play a role in asthma and allergic inflammation. Immunohistochemistry analysis showed that cytosolic DNA accumulated in airway epithelial cells isolated from mouse models of acute asthma and allergic airway inflammation [310]. Cytosolic DNA and cGAS were found co-localized in human bronchial cells treated with IL-33, an inflammatory cytokine involved in the asthma attack. This phenomenon was dampened in cells pretreated with the mitochondrial antioxidant MitoTEMPO. Similarly, genetic deletion of cGAS in Clara cells attenuated ovalbumin and house dust mite-induced mouse airway inflammation. These findings may suggest that mtROS overproduction triggers mtDNA release into the cytosol, which contributes to asthma and allergies.

### AIM2/NLRP3 inflammasome

Release of mtDNA and activation of the AIM2/NLRP3 inflammasome have been suggested to play a role in acute respiratory distress syndrome (ARDS) and acute lung injury (ALI). ARDS is characterized by acute inflammation and is common in the lungs of COVID-19 patients. It has been shown that metformin, by inhibiting cI of the ETS, decreased OXPHOS and ATP production, reducing mtDNA synthesis and generation of ox-mtDNA [311]. By doing so, it prevented NLRP3 inflammasome activation by ox-mtDNA in macrophages and myeloid cells, protecting mice from LPS-induced ARDS. Similarly, the activation of the NLRP3 inflammasome by mtDNA was also shown in a mouse model of ALI induced by LPS [312]. Authors showed that macrophages treated with LPS increased OxStr and cytosolic levels of mtDNA, with cGAS directly activated by cytosolic mtDNA, and NLRP3 inflammasome indirectly activated by STING cross-signaling. As a proof of concept, *cGas* or *Sting* KO mice experienced an attenuated LPS-induced ALI. In a similar mouse model of sepsis-associated ALI, Huang and colleagues (2023) showed that the ccf-mtDNA level was high in the BAL, and it activated M1 alveolar macrophages [313]. Interestingly, they also generated a hybrid protein composed of recombinant DNase I and human serum albumin to target pulmonary ccf-mtDNA. The delivery of this inhalable protein enhanced the therapeutic effect of DNase I, attenuated mouse lung inflammation and injury, and improved survival to sepsis, providing evidence that digestion of extracellular mtDNA could be used as a potential therapy to blunt lung inflammation.

### TLR9

Mitochondrial dysfunction and impaired mitophagy are hallmarks of COPD, IPF, and other lung diseases [314], supporting the possibility that mtDNA mislocalizes and triggers the RAGE-TLR9 pathway.

COPD is characterized by persistent lung inflammation, mostly caused by prolonged exposure to CS [314, 315]. Tobacco smoke contains RAGE ligands and induces TLR9 expression in CD8 + T cells, which release pro-inflammatory cytokines that contribute to COPD pathogenesis [316, 317]. TLR9 engagement appears to be the primary pro-inflammatory pathway, as *Tlr9* KO mice did not develop COPD after chronic exposure to CS [318]. We detected high levels of ccf-mtDNA in the plasma of COPD patients, serum of mice, and conditioned medium of cells exposed to CS [132]. A recent investigation from our lab and another independent study corroborated these results in a bigger cohort and found that elevated levels of ccf-mtDNA in the plasma were associated with mild and moderate COPD, with high mtDNA levels predicting COPD exacerbations [319, 320]. Altogether, these findings suggest that ccf-mtDNA may trigger the RAGE-TLR9 cascade in COPD.

Ccf-mtDNA levels were also found elevated in the plasma of the IPF patients and able to predict acute exacerbation and death [308, 321]. Interestingly, Bueno et al. (2019) reported that ccf-mtDNA levels in plasma were inversely correlated with PINK1 expression in the lungs of IPF patients [100]. They showed that AECII—responsible for the pro-fibrotic signaling in IPF—internalized extracellular mtDNA by endocytosis, increasing IL-6 and TGF-β secretion mediated by TLR9 and NF-κB signaling. As PINK1 overexpression inhibited the secretion of pro-inflammatory cytokines [100], these results highlight the link between dysfunctional mitophagy, mtDNA release, and TLR9 driving inflammation in IPF.

Intratracheal instillation of silica in mice induced pulmonary inflammation driven by neutrophil recruitment, caused by the release of mtDNA from necrotic cells, which activated the TLR9 signaling [322]. A recent study links the ccf-mtDNA and ox-mtDNA to the upregulation of lysine-specific demethylase jumonji domain-containing protein 3 (JMJD3), a protein promoting the expression of inflammatory genes [323]. The authors showed that ccf-mtDNA and ox-mtDNA are high in the serum of patients with acute pancreatitis. Pancreatic necrotic cells release mtDNA that engage TLR9 and STING, increasing the expression of JMJD3 in monocytes and inducing pancreatitis-associated lung inflammation. This finding highlights the role of ccf-mtDNA as a pro-inflammatory signal involved in interorgan communication.

## mtDNA signaling via DSRs in neurodegenerative diseases

Neurodegenerative diseases are heterogeneous neurological disorders affecting memory, cognition, and sensory and motoric function [324]. Neurons are one of the most energetically demanding cells, and mitochondrial dysfunctions contribute to neurodegenerative diseases with mtDNA engaging DSRs and causing neuroinflammation [325].

### cGAS-STING

Specific mutations of Parkin and PINK1 (Sect. "Dysfunctional autophagy, mitophagy, remodeling of the mitochondrial membranes, and budding of mitochondrial-derived vesicles") are associated with Parkinson’s disease (PD), with the serum of these patients containing high levels of pro-inflammatory cytokines [99, 326, 327]. Similarly, acute mitochondrial stress induced by exhaustive exercise in *Pink* or *Parkin* KO mice and chronic stress induced by the accumulation of mtDNA mutations in mutator mice led to IFN-I expression [99]. In these mice, mtDNA was released into the serum and triggered the IFN-I response by the STING pathway, probably mediated by cGAS. The concurrent loss of STING in *Parkin* or *Pink* KO mice rescued motor defects and prevented degeneration of dopaminergic neurons. These findings link mtDNA leakage induced by defective mitophagy to STING-mediated neuroinflammation in PD.

Other proteins have been noticed to increase cytosolic or ccf-mtDNA levels in several neurogenerative models. These include the arylalkylamine N-acetyltransferase (AANAT), caseinolytic mitochondrial matrix peptidase proteolytic subunit (CLPP), human ortholog of yeast mitochondrial AAA metalloprotease (YMEL1), and the double-stranded RNA-specific endoribonuclease (DICER1). In the brain of a mouse model of Huntington’s disease, the level of AANAT, an enzyme involved in the synthesis of melatonin—an endogenous ROS scavenger synthesized by the mitochondria – was decreased [328]. The cortical neurons lacking AANAT showed OxStr, decreased ∆Ψ_mt_, increased cytosolic mtDNA levels, and activated cGAS-STING-IRF3. *Aanat* KO neurons depleted of mtDNA or transfected with DNase I attenuated inflammatory markers, confirming that cytosolic mtDNA was involved in the inflammatory response.

CLPP is a mitochondrial serine protease responsible for the degradation of damaged and misfolded proteins. *Clpp* KO mice are affected by growth retardation, deafness, and showed altered immune response with high levels of IFN-I. West’s group observed that Clpp-deficient MEFs exhibited altered nucleoid morphology, mtDNA instability, and increased mtDNA release into the cytosol [329]. The ablation of cGAS or STING, or the depletion of mtDNA, decreased IFN-I expression, indicating that mtDNA engaging cGAS-STING-IRF3 stress was driving the IFN-I response. These findings may translate into therapy for the Perrault syndrome, a disease caused by CLPP mutations.

The ATP-dependent proteolytic complex YMEL1 is localized to the IMM and coordinates mitochondrial dynamics and biogenesis by regulating fusion and fission [330]. Mutations in YMEL1 cause neurological disorders and motor delay. Accordingly, neuron-specific *Yme1l* KO mice experience ocular dysfunction and retinal inflammation. The loss of YMEL1 in mouse retinal cells and MEFs increased the stability and half-life of the pyrimidine nucleotide carrier SLC25A33 [330]. In turn, increased SLC25A33 protein deregulated mitochondrial nucleotide uptake, inducing an imbalance of the nucleotide pool and release of mtDNA into the cytosol to replenish the cytosolic nucleotide pool [72]. The presence of cytosolic mtDNA increased ISG expression by the cGAS-STING pathway. These findings revealed a tight crosstalk between mtDNA stress caused by nucleotide imbalance and cGAS-mediated immune response via cytosolic mtDNA.

Macular degeneration (blindness) is caused by the death of retinal pigmented epithelial cells. These cells support photoreceptors, specific neurons that convert light into nerve impulses. The disease is associated with DICER1 deficiency, a ribonuclease III that cleaves double-stranded RNA. DICER1 deficiency leads to the accumulation of *Alu* mobile element RNA transcripts and the activation of the caspase-4/NLRP3 inflammasome. Kerur and colleagues (2018) discovered that this noncanonical inflammasome depended on the IFN-β expression induced by cGAS signaling. Interestingly, cGAS was activated by mtDNA released into the cytosol through the mPTP [331].

Recently, Ablasser’s group found that in aged mice, cGAS-STING signaling induced the aging-related IFN-I response in microglia, causing neuronal loss and cognitive impairment [74]. Mechanistically, they demonstrated that warped aged mitochondria release mtDNA in the cytosol by mPTP/VDAC oligomerization, which in turn activates cGAS-STING signaling. As proof of concept, inhibition of VDAC oligomerization by VBIT-4 suppressed the IFN-I response. In aged mice, pharmacological inhibition of STING by its antagonist H-151 decreased immune markers of aging, improving memory. Similarly, in the retina—one of the most vulnerable part of the central nervous system (CNS)—aged mice accumulate mitolysosomes [101]. This promotes the cytosolic release of mtDNA, cGAS-STING cascade with the upregulation of IFN-I response and inflammation. Boosting mitophagy by urolithin A injections in old mice, curtailed cGAS/STING activation, decreasing inflammation [101].

### AIM2/NLRP3 inflammasome

The activation of NLRP3 inflammasome by mtDNA has been proposed but not demonstrated in microglia and neurons of a rat model of cerebral I/R, PD, and Alzheimer’s disease (AD) [332–334]. Similarly, it has been suggested that anxiety, memory, and the regulation of neuronal morphology are influenced by AIM2 inflammasome activation triggered by dsDNA [335]. IFI16 (same family of AIM2, Sect. "AIM2 inflammasome") and cytosolic dsDNA proximal to the mitochondria, accumulated in the brain of patients with PD [248]. Furthermore, neuroblastic cells with autophagic defects exhibit high levels of cytosolic mtDNA and IFN-I, which were rescued by the overexpression of DNase II or depletion of IFI16. These results were recapitulated in vivo using a zebrafish model of PD, suggesting a role of mtDNA in engaging IFI16. Overall, further research is needed to verify whether mtDNA docks AIM2 and NLRP3 inflammasomes in neurodegenerative diseases.

### TLR9

Several conflicting studies on the role of ccf-mtDNA were reported in PD and AD, pointing out the need for future investigations to determine its interaction with TLR9. Low levels of ccf-mtDNA have been detected in the cerebrospinal fluid (CSF) of patients with sporadic PD as well as familial and sporadic AD [336, 337]. The low ccf-mtDNA could reflect the decreased mitochondrial biogenesis and mtDNA copy number observed in the affected neurons [338, 339]. Another hypothesis is that decreased ccf-mtDNA levels in the CSF could be influenced by comorbidities and medical interventions, as shown in the CSF of PD patients [340]. On the contrary, patients affected by multiple sclerosis or other CNS disorders driven by inflammation, showed elevated levels of ccf-mtDNA in the plasma [341]. Increased ccf-mtDNA levels in the serum have been also observed in children affected by autism spectrum disorder, in people who have attempted suicide, and in patients with several physiological states [342]. While the contribution of TLR9 to the inflammation observed in neurodegenerative and psycho/neuroendocrinal conditions requires further investigation, it is reasonable to assume that the elevated ccf-mtDNA levels reported in these studies could trigger inflammation by TLR9 [342].

## mtDNA signaling via DSRs in viral and bacterial infections

Several viral and bacterial infections cause mitochondrial dysfunction that leads to mtDNA leakage into the cytosol and circulation. Dengue (DENV), Zika, influenza viruses, Kaposi’s sarcoma-associated herpesvirus (KSHV), *Mycobacterium tuberculosis (Mtub)* have been shown to contribute to mtDNA release triggering an immune response by cGAS, AIM2/NLRP3, and TLR9.

### cGAS-STING

Elevated ccf-DNA levels were found in the serum of patients with Dengue fever [343]. Based on this observation, A549 and THP1 cells were studied upon infection with a DENV serotype 2 vaccine strain [171]. Infected cells exhibited increased cytosolic mtDNA levels that activated the cGAS cascade. The resulting innate immune response limited the viral spread to adjacent uninfected cells. Although the mechanism by which mtDNA was released into the cytosol was not investigated, two reports showed that the C-terminus of the DENV M protein decreases ΔΨ_mt_, inducing MOMP [170, 344]. Other studies demonstrated that the DENV NS2B3 protease cleaves MFN1 and -2, altering mitochondrial dynamics [345, 346]. Reasonably, both mechanisms could contribute to mtDNA release. Paradoxically, it has been shown that DENV NS2B targets cGAS for lysosomal degradation, preventing its mtDNA detection and IFN-I expression in infected cells, to evade immune response [347]. Zika virus belongs as DENV to the same *Flaviviridae* family. It promotes mtDNA release into the cytosol of infected cells, and its non-structural protein S1 triggers cGAS cleavage to avoid antiviral response and favour NLRP3 inflammasome [348]. Viroporin activity of the M2 protein of the influenza virus and the 2B protein of the encephalomyocarditis virus (EMCV) act with similar mechanisms [169].

Cells infected with Kaposi’s sarcoma-associated herpesvirus (KSHV) showed increased levels of cytosolic mtDNA and cGAS-STING-IRF3 activation. Furthermore, infected cells released EVs containing mtDNA that act like long-way messengers to trigger an antiviral response and favor the survival of uninfected cells [349]. Recently, it has been also shown that vesicular stomatitis virus (RNA virus) or herpes simplex virus 1 (DNA virus) activate the nuclear respiratory factor-1 (NRF-1, regulator of the mitochondrial biogenesis) to antagonize antiviral immunity [350]. Myeloid-specific NRF-1 deficient mice showed aggravated virus-induced mitochondrial damages with high levels of cytosolic mtDNA and IFN-I transcripts. This phenotype was probably driven by the cGAS-STING pathway because blocking mPTP by CsA inhibited the mtDNA release, attenuating the immune response.

Mislocalized mtDNA is an immune signal also during bacterial infection. Despite years of vaccination and drug therapy, tuberculosis (TB) remains one of the top infectious killer worldwide. Once engulfed by a macrophage, the *Mtub* activates the ESAT-6 secretion system-1 (ESX-1) to rupture the phagosome and spread into the cytosol [351]. Ablasser’s group demonstrated that macrophages infected with *Mtub* mounted an IFN-I response by cGAS signaling [352]. *Mtub* promoted IFN-I secretion in the early phase of infection by several strain-dependent mechanisms, including the release of host mtDNA, but not bacterial DNA, into the cytosol [353]. Similarly, macrophages infected by *Mycobacterium marinum* lose phagosomal membrane integrity, with bacterial DNA leaking from the disrupted phagosome unable to trigger IFN-I signaling by cGAS. On the contrary, ESX-1 decreasing ΔΨ_mt_ caused OMM rupture and mtDNA leakage into the cytosol, with consequent cGAS-mediated IFN-I expression [354].

MtDNA-induced cGAS activation is also induced by *Mycobacterium abscessus (Mabs),* which is responsible for many nosocomial infections and is particularly harmful in patients with lung diseases [355, 356]. A highly virulent variant of *Mabs* increased mtROS and ox-mtDNA in infected murine macrophages [357]. Ox-mtDNA was released into the cytosol and promoted the cGAS-dependent IFN-I expression, together with the NLRP3-dependent IL-1β expression. As proof of concept, these effects were mitigated by treatment with mitoTEMPO and the mPTP-opening inhibitor CsA.

### AIM2/NLRP3 inflammasome

Infection with severe fever with thrombocytopenia syndrome virus (SFTSV) induced oxidation and release of mtDNA into the cytosol via BAX/BAK, activating NLRP3 inflammasome [358]. Similar results were found in a model of infection of rift valley fever virus [359]. Recently, Wallace’s group showed that two viroporins of SARS-CoV-2 increased mtROS, leading to mtDNA release by mPTP and secretion of IL-1β via NLRP3 inflammasome in THP-1 cells [360]. This response was absent in cells depleted of mtDNA, decreasing mtROS or blocking mPTP.

Immune reconstitution inflammatory syndrome (IRIS) is a common complication caused by antiretroviral therapy in patients co-infected with HIV and TB [361]. Patients with IRIS mount an excessive inflammatory response to opportunistic pathogens with elevated ccf-mtDNA and IL-18 plasma levels, high AIM2 and NLRP3 expression, and caspase-1 activation in their monocytes [362]. These results suggest that ccf-mtDNA may activate the AIM2/NLRP3 inflammasome in IRIS. Recently, it has been also shown that mitoribosome-targeting antibiotics mitigate NLRP3 inflammasome activation by inhibiting mtDNA oxidation and release [363], advocating for their therapeutic application during viral and bacterial infections.

### TLR9

It has been already described that DENV activates a cGAS-mediated immune response [171]. Additionally, DENV infection caused mPTP opening and release of mtDNA into the cytosol of DCs with consequent activation of TLR9 [364]. Lately, Lai et al*.* (2021) showed that the mitochondrial cytidine/uridine monophosphate kinase 2 (CMPK2) was upregulated in BMDMs and DCs infected with DENV [365]. *Cmpk2* KO cells, upon DENV infection, decreased mtDNA levels into the cytosol, TLR-9 activation, and IFN-α transcripts, indicating that CMPK2 has an antiviral role, linking mtDNA and TLR9 pathway.

Patients with septic shock caused by multidrug-resistant bacteria had higher levels of ccf-mtDNA compared to healthy controls [366]. Furthermore, patients with end-stage illness caused by infections displayed elevated ccf-mtDNA levels compared to those discharged from the ICU [366]. Similar findings were shown in COVID-19 patients. Baseline levels of ccf-mtDNA in the plasma were higher in COVID-19 patients compared to healthy controls [367], and even higher in patients who died or required ICU admission, with ccf-mtDNA levels correlating with a poor prognosis [115]. While it is widely established that TLR9 recognizes bacterial and viral DNA motifs, the role of ccf-mtDNA in activating TLR9 during these infections is not yet fully elucidated. To this purpose, Mangalmurti’s group showed that mtDNA bound to TLR9 on RBCs is elevated in patients with sepsis and COVID-19 [259]. Using a mouse model, they demonstrated that CpG-mtDNA binding to TLR9 induced morphological changes of RBCs, which promoted erythrophagocytosis with spleen congestion, and triggered innate immunity by increasing the IFN and IL-6 transcripts [259]. These findings suggest that ccf-mtDNA levels may be a useful prognostic tool for some bacterial and viral infections.

## Strategies to analyze mtDNA release and its binding to DSRs

To comprehend where/how mtDNA activates immune responses, proper tools are required to analyze mtDNA release and its binding to DSRs. There are many approaches to study mtDNA release and the biological cascades triggered by its mislocalization. ROS inducers, protonophores, toxin-like ionophores, and ER-stressing compounds are the most used treatments to activate mtDNA release (Sects. "Inducers of reactive oxygen species"-"Protonophores, toxin-like ionophores, and ER-stressing compounds"). Meanwhile, antioxidants and mPTP blockers are widely used as inhibitors of mtDNA release (Sects. "Antioxidants"-"Inhibition of mPTP and VDAC oligomerization"). Furthermore, to understand the biological cascades triggered by “mislocalized” mtDNA, we report several strategies to activate mtDNA-sensing pathways (Sect. "Strategies to activate mtDNA-sensing pathways") or to prevent the binding of mtDNA to DSRs (Sect. "Strategies to prevent the binding of mtDNA to DSRs").

### Activators of mtDNA release

#### Inducers of reactive oxygen species

As discussed above, O_2_^−^, OH^−^, and H_2_O_2_ oxidize lipids and proteins of the IMM and OMM, altering MMP and favouring mtDNA release [52, 53, 166]. Specific ETS inhibitors, such as rotenone and other toxic compounds like heavy metals, increase mtROS production, especially O_2_^−^, and are commonly used to trigger mtDNA release into the cytosol (Table 3) [268–270].

#### Protonophores, toxin-like ionophores, and ER-stressing compounds

Drugs and toxins that directly permeabilize the IMM facilitate mtDNA release into the cytosol (Table 3). These include the uncoupling agents carbonylcyanide-4-trifluoromethoxyphenylhydrazone (FCCP) and carbonylcyanide-3-chlorophenylhydrazone (CCCP), lipid-soluble weak acids that increase IMM permeability to the hydrogen ions, resulting in decreased ΔΨ and increased mtROS production [275, 276].

**Table 3:** Summary of the main inducers of mtDNA release and strategies to activate the DNA-sensing receptors

Molecule	Target	Mechanism	Reference
ABT-737	OMM permeabilization	Low dose (1–5 µM) fosters mitochondrial release without triggering cell death (“Minority MOMP”)	[83, 89, 267]
Dideoxycytidine (Zalcitabine)	TFAM	It promotes TFAM degradation, mtDNA instability and release into the cytosol	[130]
Doxorubicin	mtDNA	It promotes the release of Z-form mtDNA into the cytosol	[40]
Reactive oxygen species (by cI, cIII, bacteria)	IMM	They oxidize cardiolipin and phosphatidylethanolamine and have pleiotropic effects on mitochondrial permeability (Ca^2+^ and MOMP regulation)	[166]
Rotenone and heavy metals	Inhibits cI and increases ROS production	They oxidize cardiolipin and phosphatidylethanolamine and have pleiotropic effects on mitochondrial permeability (Ca^2+^ and MOMP regulation)	[268–270]
Thapsigargin	ER	It inhibits the SERCA ATPase channel and blocks Ca^2+^ uptake into the ER, decreasing the ΔΨ	[271–273]
Tunicamycin	ER	It inhibits N-linked glycosylation, inducing protein misfolding and increases ROS	[274]
Uncoupling agents (FCCP, CCCP)	IMM permeabilization	They dissipate the ΔΨ and increase ROS	[275, 276]
Genetic ablation of DNase II	DNA in autophagosomes	It increases DNA degradation in autophagosomes	[94]
Transfection of synthetic or exogenous DNA/mtDNA	DSRs	DNA binds directly the DSRs	[14, 93, 186, 188, 277, 278]

ABT-737, the first drug developed for cancer chemotherapy, inhibits Bcl-2 and Bcl-xL. At low (1–5 µM) concentrations, it causes mtDNA release by promoting BAX/BAK oligomerization and MOMP formation [14, 101, 368] without triggering apoptosis [83, 89, 267]. Stresses targeting ER have also been shown to induce mtDNA release into the cytosol. Several techniques are available to stimulate ER stress. A common approach is treatment with thapsigargin. By inhibiting the sarcoplasmic reticulum Ca^2+^ ATPase (SERCA), thapsigargin blocks Ca^2+^ uptake into the ER. As a result, Ca^2+^ accumulates in the cytosol and mitochondria. Mitochondrial Ca^2+^ overload drastically decreased ΔΨ_mt_, causing the release of mitochondrial contents into the cytosol, including mtDNA [271–273]. Tunicamycin induces ER stress by preventing N-linked glycosylation of nascent polypeptides, blocking protein folding and transit. Treatment with tunicamycin has been also shown to cause mtDNA release into the cytosol of BMDMs [274].

### Strategies to activate mtDNA-sensing pathways

The major strategies used to investigate mtDNA activation of DSRs include **(**Table 3**)**: i) the transfection of exogenous mtDNA or a similar synthetic DNA (poly(dA:dT) [14, 93, 186, 188, 277, 278]; ii) the genetic or pharmacological downregulation of TFAM to promote mtDNA instability [15, 37, 309]; iii) the genetic ablation of DNase II to prevent the digestion of mtDNA in the autophagosomes [94].

For instance, it has been shown that zalcitabine (ddC) induced mtDNA depletion by decreasing TFAM expression. The subsequent mitochondrial dysfunction increased mitophagy and stalled autophagic flux, inducing mtDNA release into the cytosol and triggering cGAS [130]. This process was prevented by inhibiting TFAM degradation using 2,3,5,6-tetramethylpyrazine (TMP) or by inhibiting mitochondrial Lon peptidase 1 (LONP) using bortezomib. Similarly, elevated protein kinase A (PKA) activity increased TFAM phosphorylation and induced its dissociation from mtDNA, reducing its stability and promoting release into the cytosol by MOMP [364]. Treatment with H89—a PKA inhibitor—reversed these effects. Overall, these technical tricks to induce mtDNA release result in the upregulation of downstream cytokines that are mediated by the engagement of the mtDNA with DSRs.

### Inhibitors of mtDNA release

#### Antioxidants

MtDNA release into the cytosol is inhibited by treatment with mitochondrial-targeted antioxidants, which avoid DNA and lipid oxidation and mPTP opening (Table 4). Researchers often used MitoQ and MitoTEMPO to inhibit mtDNA oxidation [93, 310, 353, 357, 364]. MitoQ is a derivative of idebenone, while MitoTEMPO is an antioxidant piperidine nitroxide attached to a lipophilic triphenylphosphonium cation. These compounds pass directly through the IMM and reduce O_2_^−^.

**Table 4:** Summary of the main inhibitors of mitochondrial DNA release and strategies to inhibit its binding to the DNA-sensing receptors

Molecule	Target	Mechanism	Reference
BAI1	BAX/BAX	It prevents BAX/BAK translocation and oligomerization	[88]
BAPTA-AM and Minocycline	mPTP	They are Ca^+2^ chelators that inhibit mPTP opening	[59, 169, 364]
Carbon monoxide	ROS	It inhibits mitochondrial superoxide overproduction and indirectly sustains ∆Ψ_mt_	[275]
CsA and NIM811	mPTP	They prevent mPTP opening by inhibiting the interaction of CypD with the mPTP	[59, 60, 62, 93, 169, 331, 360, 364, 369]
^a^Dideoxycytidine	Specific POLG inhibitor	It inhibits mtDNA replication, removing the substrate (mtDNA) required to bind the DSRs	[14, 15, 267, 370]
EGCG/NAC/Riboflavin	ROS	They are broad-target antioxidants that decrease ROS and prevent mtDNA oxidation	[269, 371–374]
Ethyl pyruvate	ROS	It attenuates mitochondrial damage by decreasing ROS	[375, 376]
GW4869	Exocytosis	It inhibits exocytosis	[131, 133]
Metformin	cI	It (1–10 mM) inhibits cI, decreases ROS production and mtDNA release	[373]
MitoQ and MitoTEMPO	mPTP	They are mito-targeted antioxidants that reduce superoxide and prevent lipid peroxidation and mPTP opening	[93, 310, 353, 357, 364]
TMP (2,3,5,6-tetramethylpyrazine) and Bortezomib	TFAM	They inhibit TFAM degradation and indirectly increase mtDNA stability	[278]
VBIT-4	VDAC	It inhibits the oligomerization of VDAC and mPTP opening	[62, 72–74]
Xanthohumol	SIRT-1	It reduces ROS production by SIRT-1 signaling	[377]
3-methyladenine	Autophagy	It inhibits autophagy	[134]
DNase I or II overexpression or exogenous treatment	mtDNA	They degrade the substrate (mtDNA) required to bind the DSRs	[93, 213, 248, 268, 277, 278, 373]
mtDNA depletion by ethidium bromide	mtDNA	It decreases the substrate (mtDNA) required to bind the DSRs	[14, 93, 130, 267, 277, 331]
Transient (siRNA) or stable (KO) downregulation of DSRs	DSRs	They decrease/nullify DSRs avoiding the docking with mtDNA	[93, 277]
RU.52, G140	cGAS	They are inhibitors of cGAS	[39, 101, 109, 378]

^a^Of note, dideoxycytidine has been also reported to promote TFAM degradation, mtDNA instability and release into the cytosol, acting as a promoter of mtDNA release [130]

Non-mitochondrial-targeted antioxidants can also prevent mtDNA oxidation and release. Epigallocatechin gallate (EGCG) is a polyphenol that has been shown to attenuate ROS production. It prevented mtDNA oxidation and its binding to NLRP3 in BMDMs in an in vitro model of acute gout and a model of lung injury [371, 372]. Similarly, N-acetylcysteine (NAC) decreased mtDNA release from ADP-activated platelets and inhibited caspase-1 activation induced by rotenone in BMDMs [269, 373]. Riboflavin prevented mtROS production and mtDNA release, attenuating NLRP3 inflammasome assembly in macrophages [374].

In a model of thrombosis, (0.5 M) xanthohumol attenuated ROS production through a sirtuin-1-dependent mechanism, inhibiting mtDNA release and platelet activation [377]. The same effect was found by using low doses of metformin (1–10 mM) that inhibited cI, decreased ROS overproduction and lipid peroxidation, avoiding mtDNA release [373]. On the contrary, high concentrations of metformin (> 100 mM) increased mtDNA release.

In a study, carbon monoxide (CO) was used to inhibit mtROS production, preserving the ΔΨ_mt_, avoiding mtDNA release into the cytosol and the activation of NLRP3-inflammasome [275]. Similarly, ethyl pyruvate, another ROS scavenger, attenuated mitochondrial damage, decreased mtDNA and HMGB1 release, and inhibited NLRP3 inflammasome activation [375, 376]. Even though it has not proved yet, it is reasonable to believe that overexpressing endogenous antioxidant enzymes (SOD2, glutathione peroxidase 4, peroxiredoxin 3) or xenotopic mitochondrial alternative enzymes (alternative oxidase, NADH dehydrogenase NDX and Ndi1) [379, 380] could be beneficial to avoid mtDNA release.

#### Inhibition of mPTP and VDAC oligomerization

Another strategy to inhibit mtDNA release and investigate its role in the activation of immune cascades is the inhibition of mPTP opening (Table 4). It has been demonstrated that ssDNA and dsDNA cross IMM and OMM via mPTP [56, 64, 65]. The mitochondrial matrix protein CypD directly regulates mPTP opening by binding to its pore components [381, 382]. CsA is a non-ribosomal peptide and immunosuppressant that inhibits mPTP opening by preventing its interaction with CypD [60, 369]. CsA is often used to investigate how mtDNA release from the mitochondria can be prevented by mPTP inhibition [93, 331]. NIM811 works similarly to CsA, and as minocycline and BAPTA-AM (Ca^+2^ chelators), inhibit mPTP opening and prevent mtDNA release [59, 169, 360, 364]. Another routinely used strategy to inhibit mtDNA release is to block the oligomerization of VDAC by VBIT-4 [62, 72, 74] (Table 4).

### Strategies to prevent the binding of mtDNA to DSRs

To study the consequences of mtDNA release, researchers prevent its binding to DSRs by: i) using mtDNA-depleted cells (ρ^0^) [130, 331, 360]; ii) decreasing total mtDNA content by treating cells with low concentrations of ethidium bromide [14, 93, 130, 267, 277]; iii) inhibiting mtDNA replication with ddC, a specific POLG inhibitor [14, 15, 130, 267, 370] (Table 4). DNase I or II overexpression or treating the extracellular milieu with DNase I have been useful techniques to investigate how the digestion of mtDNA suppresses the activation of specific DSRs, indicated by the downregulation of downstream cytokines [93, 213, 248, 268, 277, 278, 373]. Another strategy is to downregulate or deplete DSRs by transient (siRNA) or stable (KO) genetic manipulation, which abolishes their downstream signaling [93, 277], or to inhibit DSRs by drugs like RU.521 or G140 for cGAS [39, 101, 109, 378].

## Conclusions and perspective

In this comprehensive review, we emphasized the role of mislocalized mtDNA. While the mechanisms that promote mtDNA release into the cytosol and extracellular compartments are becoming clear, studies in the last 15 years have revealed that mtDNA can initiate an inflammatory response by binding to several DSRs. MtDNA should be considered not only as a marker of mitochondrial dysfunction but also as an autocrine and paracrine signal (mitohormone) involved in the immune signaling between cells and organs. We have described the causes and consequences of mtDNA mislocalization and summarized the recent findings and gaps in vascular and metabolic, kidney, lung and neurodegenerative diseases as well as viral and bacterial infections. We also condensed the common strategies used to induce or inhibit mtDNA release and DSRs.

Based on the current state of the field, to better understand the role of mtDNA release in pathophysiology, we propose that the following factors should be considered during future experimental design:

i) the mechanism by which mPTP enables mtDNA release is still unknown [62]. Similarly, it remains unclear how the IMM is permeabilized, allowing mtDNA release during IMM herniation and MOMP [84, 85]. These gaps call for future studies on the structural and functional characterization of the mPTP and IMM permeabilization. Additionally, because mitochondrial nucleoids are tethered to the IMM by DNA–protein interaction and protein–protein interactions (for example by PHB1 or MICOS complex) [62, 383–385], it will be essential to address the role of the nucleoid-tethering proteins in the mtDNA release.

ii) both mtDNA and nDNA should be detected by a duplex TaqMan qPCR reaction using primer sets specific to each genome to decipher the single contribution [386], since mitochondrial dysfunction could also induce genome instability [11, 234]. Absolute values of mtDNA and nDNA levels should be quantified by digital PCR. They should be expressed as copies per number of cells, copies per amount of proteins, copies per amount of tissue for the measurement of DNA in the cytosolic fraction. Copies per unit of volume (copies/ml or copies/µl) or per number of vesicles (nanoparticle analysis) should be reported for the levels of ccf-mtDNA.

iii) qualitative analysis of the released mtDNA should be conducted. The oxidation and fragmentation state of mtDNA, together with its sequencing, could also shed new insight into its specific role.

iv) cytosolic and/or extracellular mtDNA should be spatiotemporally detected by fractionation and microscopy using specific dyes [387, 388]. MtDNA should be contextualized to its localization in autophagosomes, vesicles, and PM.

v) mtDNA release should be confirmed by genetics and/or pharmacological inhibition of major routes (Sects. "Mechanisms that lead mtDNA release into the cytosol"-"Mechanisms that lead mtDNA release into extracellular environments").

vi) mtDNA binding to DSRs should be verified. To achieve this goal, it would be beneficial to employ co-immunoprecipitation and/or in situ proximity ligation assay.

vii) the binding of mtDNA to new and/or multiple DSRs should be considered. To discover new pathophysiological mechanisms and address specific therapies, the binding partners of mtDNA need to be characterized. For example, the recent findings that mt-Z-DNA stabilizes ZBP1, make ZBP1 a novel cytoplasmic DSR for the mtDNA [40]. The binding of mtDNA to ZBP1 [193, 389, 390] or IFI16 (Sect. "AIM2 inflammasome") [248, 249] and their potential as a therapeutic target remains explored in a few pathological conditions, calling for further analyses. Considering the same disease, multiple DSRs are triggered by mtDNA. For instance, mtDNA activates cGAS [391] and TLR9 [322] in silica-induced-lung inflammation; cGAS [392] and TLR9 [100] in lung fibrosis; cGAS [303] and TLR9 [304] in AKI; cGAS [37] and AIM2 [305] in CKD. This rationale calls for new studies, to understand whether an activated DSR is more important than the others for its stoichiometric and conformational features, or its specific localization within the cell. These studies should use cells and animal models of double/triple KO for the DSRs, and they should consider the cross signaling between DSRs activated pathways.

viii) new molecular tools to explore the release of mtDNA and its binding to DSRs should be generated. Engineered mtDNA or DSRs tagged with fluorescent probes could be used to show direct evidence of binding in vivo by intravital microscopy. Additionally, time-course experiments could reveal important windows for pharmacological treatment.

## Data Availability

No datasets were generated or analysed during the current study.
